# *Ustilago maydis* natural antisense transcript expression alters mRNA stability and pathogenesis

**DOI:** 10.1111/mmi.12254

**Published:** 2013-05-30

**Authors:** Michael E Donaldson, Barry J Saville

**Affiliations:** 1 Environmental and Life Sciences Graduate ProgramPeterborough, ON, Canada, K9J 7B8; 2 Forensic Science Program, Trent UniversityPeterborough, ON, Canada, K9J 7B8

## Abstract

*Ustilago maydis* infection of *Zea mays* leads to the production of thick-walled diploid teliospores that are the dispersal agent for this pathogen. Transcriptome analyses of this model biotrophic basidiomycete fungus identified natural antisense transcripts (NATs) complementary to 247 open reading frames. The *U. maydis* NAT cDNAs were fully sequenced and annotated. Strand-specific RT-PCR screens confirmed expression and identified NATs preferentially expressed in the teliospore. Targeted screens revealed four *U. maydis* NATs that are conserved in a related fungus. Expression of NATs in haploid cells, where they are not naturally occurring, resulted in increased steady-state levels of some complementary mRNAs. The expression of one NAT, *as-um02151*, in haploid cells resulted in a twofold increase in complementary mRNA levels, the formation of sense–antisense double-stranded RNAs, and unchanged Um02151 protein levels. This led to a model for NAT function in the maintenance and expression of stored teliospore mRNAs. In testing this model by deletion of the regulatory region, it was determined that alteration in NAT expression resulted in decreased pathogenesis in both cob and seedling infections. This annotation and functional analysis supports multiple roles for *U. maydis* NATs in controlling gene expression and influencing pathogenesis.

## Introduction

The ‘true smut fungi’ include over 1600 fungal plant parasites. The common name ‘smut’ originates from the sooty appearance of the large numbers of melanized teliospores produced in the plant during infection. These are the dispersal agents of the fungus (Begerow *et al*., 2006). The majority of smuts infect members of the Cyperaceae (the sedges; Vanky, 2002) and the Poaceae (the grasses), which include economically important cereal crops such as barley, oats, wheat, corn and sorghum (Martinez-Espinoza *et al*., 2002). *Ustilago maydis* (DC) Corda, the causal agent of ‘common smut of corn’, has become a valuable model for studying biotrophic fungal plant-pathogen interactions.

*Ustilago maydis* can infect corn (*Zea mays* L. ssp. *mays*), and its ancestor, teosinte (*Z. mays* L ssp. *parviglumis*). Disease symptoms include chlorosis and the formation of tumours on the stems, leaves, tassels and ears (reviewed in Banuett, 1995). In its non-pathogenic form, this fungus grows as a budding saprophytic haploid. The pathogenic cycle is initiated by the fusion of compatible haploids to form an infectious dikaryon. Haploid cell fusion and sexual reproduction are controlled by the *a* and *b* mating type loci, where compatibility is governed by the presence of different alleles for both loci (reviewed in Banuett, 1995; Kahmann and Kämper, 2004). Dikaryotic mycelia penetrate the plant surface using specialized structures called appressoria, and subsequently grow as obligate biotrophs, between and through plant cells (Snetselaar and Mims, 1992; Banuett and Herskowitz, 1996). In response to fungal infection, tumours develop, within which *U. maydis* undergoes karyogamy and hyphal fragmentation, leading to the formation of thick-walled dormant teliospores (Snetselaar and Mims, 1994; Banuett and Herskowitz, 1996). The tumours dry out and crack, leading to the dispersal of teliospores, which can remain dormant for years (Christensen, 1963). Teliospore germination and meiosis are temporally linked (reviewed in Saville *et al*., 2012), producing haploid sporidia which can initiate new rounds of infection.

The numerous characteristics that have established *U. maydis* as the model biotrophic fungal plant pathogen have been well reviewed (Banuett, 1995; Bölker, 2001; Martinez-Espinoza *et al*., 2002; Basse and Steinberg, 2004; Kahmann and Kämper, 2004; Steinberg and Perez-Martin, 2008; Brefort *et al*., 2009; Dean *et al*., 2012; Djamei and Kahmann, 2012). Key features utilized in this study include: growth of haploids on defined media, induction of dikaryotic filamentous growth on media containing charcoal (Day and Anagnostakis, 1971), sexual cycle completion 14 days after seedling infection, production of teliospores following cob infection of greenhouse grown corn, and the induction of teliospore germination on a variety of mediums (Caltrider and Gottlieb, 1966). The genome of *U. maydis* has been sequenced (Kämper *et al*., 2006) as have the genomes of the related *Sporisorium reilianum* (head smut of maize and sorghum; Schirawski *et al*., 2010) and *Ustilago hordei* (covered smut of barley; Laurie *et al*., 2012). Annotated sequence information is available online at the Munich Information Center for Protein Sequences (MIPS; http://www.helmholtz-muenchen.de/en/mips/projects/fungi/index.html). This enables gene identification and comparative analyses leading to hypotheses regarding gene function. These are testable since *U. maydis* is amenable to transformation with homologous gene replacement (Yee, 1998; Kämper, 2004) and haploid solopathogenic strains have been developed to assess the impact of gene manipulations on pathogenesis, independent of mating (Bölker *et al*., 1995). Further, a variety of vectors, selectable markers and reporter constructs are available for functional studies of genes (Kojic and Holloman, 2000; Brachmann *et al*., 2004). Together, these attributes make *U. maydis* an excellent model within which to expand the investigation of eukaryotic gene functions to include antisense RNAs.

The creation of approximately 25 000 cDNA clones representing expressed transcripts from numerous developmental stages and growth in different nutritional conditions, aided in the initial *U. maydis* genome annotation (Kämper *et al*., 2006). Examination of the *U. maydis* expressed sequence tags (ESTs) uncovered natural antisense transcripts (NATs) corresponding to 247 open reading frames (Ho *et al*., 2007; Morrison *et al*., 2012). Typically, NATs are endogenous non-coding RNA sequences, complementary to protein-coding RNA sequences, which are conventionally called sense transcripts (reviewed in Vanhee-Brossollet and Vaquero, 1998). The majority of eukaryotic NATs are transcribed by RNA polymerase II, capped at their 5’ ends, and polyadenylated. Some NATs have introns removed by RNA splicing (reviewed in Munroe and Zhu, 2006; Beiter *et al*., 2009). These mRNA-like structural features led to NAT identification through cDNA library creation and EST or RNA-seq analyses in a wide variety of organisms; including species of animals, plants and fungi (reviewed in Munroe and Zhu, 2006; Faghihi and Wahlestedt, 2009; Donaldson and Saville, 2012). Yet, in fungi, determination of function is primarily limited to a handful of *Saccharomyces cerevisiae* NATs. The functions include: (i) transcription interference, (ii) chromatin remodelling, (iii) translation interference through dsRNA formation and (iv) a NAT encoding a repressor of sense transcript transcription (reviewed in Harrison *et al*., 2009; Tisseur *et al*., 2011; Donaldson and Saville, 2012). These intriguing functions have been identified by the investigation of very few fungal NATs, but thousands have been identified. The hundreds of NATs currently identified in *U. maydis* provide a library of potential functions for future investigation.

Phylogenetic and functional analyses have revealed that *U. maydis* lacks functional RNA-interference (RNAi) machinery (Nakayashiki *et al*., 2006; Laurie *et al*., 2008), making it an ideal candidate to study RNAi-independent mechanisms of antisense-mediated gene regulation in a fungal plant pathogen. An earlier study using a high-copy-number antisense transcript expression vector failed to effect levels of *pyr3* (encoding dihydroorotase) in *U. maydis* (Keon *et al*., 1999). However, endogenous antisense transcripts to the *U. maydis pyr3* have not been found. It is possible that antisense-mediated gene regulation is sequence-specific and highly regulated in *U. maydis*. This is the case with *S. cerevisiae*, where expression of exogenous antisense RNAs, complementary to different portions of sense transcripts, led to mixed results (Bonoli *et al*., 2006). To investigate NATs in *U. maydis*, three approaches were utilized: (i) a characterization of NAT features, assisted by the determination of full-length antisense sequences, (ii) a functional investigation of NATs, facilitated by the expression of antisense transcripts in haploid cells and regulatory region deletion, as well as (iii) a comparative analysis aimed at detecting similar NATs in *U. hordei*. Uncovering a function for NATs in *U. maydis* provided critical insight into the control of gene expression in a model fungal plant pathogen.

## Results

### Natural antisense transcript annotation

Examination of *U. maydis* EST libraries by Ho *et al*. (2007) and Morrison *et al*. (2012) led to the identification of 292 natural antisense transcripts (NATs) complementary to 247 of 6902 open reading frames (ORFs). These ESTs resulted from sequencing cDNA clones from their 5’ ends. In the current study, a sub-library was assembled consisting of all cDNA clones representing NATs and the resulting cDNAs were sequenced from the 3’ end of the transcript. This yielded 247 new ESTs which were submitted to GenBank (Accession Nos JZ083767 to JZ084013). The combined 539 ESTs were used to annotate *U. maydis* NATs.

The 5’ and 3’ ESTs were aligned to the genome. NATs with 5’ and 3’ sequence information, and the presence of a poly(A) tail were considered to be full-length and their features were annotated (Table S1). In this table, multiple ESTs representing antisense to a given ORF encoding sense transcript were separately recorded so that Table S1 represents all the identified full-length NATs. However, when the number of NATs representing each type of overlap was tabulated (Table 1), NATs complementary to a given sense transcript were considered distinct if they were represented in a separate cDNA library, or overlapped a distinct region of the sense transcript. Therefore, in calculations leading to the numbers in Table 1, the nine ESTs representing *as-um00133* (Table S1) were considered as four separate NATs because they were derived from four different cDNA libraries. Similarly the three ESTs representing *as-um05399* (Table S1) were scored as two separate NATs based on the overlapping region with the sense transcript. Using these criteria, 204 non-redundant NATs were found, with an average length of 803 nt, and an average NAT/ORF overlap of 626 nt. The average length of embedded NATs is slightly greater than the average NAT/ORF overlap length because some ORF encoding sense transcripts contain introns which are spanned by NATs. When looking at the different classes of NATs, 43% of the NATs were found embedded within ORFs, while 30% and 23% of NATs were complementary to the 3’ and 5’ ends of ORFs respectively (Table 1).

**Table 1:** Characteristics of sense–antisense transcript pairs

Type of NAT/ORF overlap	Number of nrNATs^a^	NAT length (nt)	Number of nrORFs	Length of NAT/ORF overlap (nt)
5’ end	47	878	46	477
Embedded	87	754	81	740
3’ end	61	756	59	574
Entire	9	1200	9	655
All	204	803	190	626

**a.** Only information gained from full-length NATs is included in this table.

NAT, natural antisense transcript; ORF, open reading frame; nr, non-redundant; nt, nucleotides.

The full-length NAT sequences were scanned for ORFs. The selection criteria included the need for an ORF to include a start and stop codon. While 64 NATs have no protein coding potential, 146 NATs contained a putative ORF 50–99 aa in length, and 71 NATs contained a putative ORF > 100 aa in length (Table S1). These putative ORFs were used to predict encoded peptides and these were inspected for secretion signals and similarity to known proteins in the NCBI non-redundant protein database. Eight NATs encode putative peptides containing secretion signals. Additionally, three NATs encode putative peptides with sequence similarity to hypothetical proteins from *Candida albicans* and *U. hordei* (Table S2).

NATs with 5’ and 3’ sequence information, and the absence of a poly(A) tail, or those NATs with 5’ sequence information alone, were not considered to be full-length and their characteristics are summarized in Table S3. Combined with the full-length NATs, these NATs were used to note the following: NATs complementary to genes encoding *U. maydis* effector proteins, NATs overlapping two transcripts, NATs overlapping sense transcript intron splice junctions, and NATs containing introns. Eight NATs were identified at seven loci that encode *U. maydis* effector proteins (Table S1, putative effector proteins in bold font). Very few NATs overlap multiple ORFs. The antisense *as01-um00047.2* and *as01-um06390* overlap the corresponding sense transcripts as well as the sense strand of *um00048* and *um06391* respectively. Similarly the 3’ end *as01-um10078* and *as01-um11010* are also antisense to *um10079* and *um11009* respectively (Table S1). In Table S1, 54 ORF encoding sense transcripts contain at least one intron. Of these, 29 have a NAT complementary to at least one splice junction; including 13 cases in which the NAT overlaps an entire intron (Tables S1 and S3). Eleven NATs contain one intron and *as04-um10027* contains two introns. The 13 NAT introns range from 97 nt to 1000 nt in length, with an average length of 299 nt (Tables S1 and S3). Nine of the NAT introns contain canonical 5'-GU/AG-3’ splice site dinucleotides and within this group, the introns contain possible CURAY or YYRAY branch point sites < 50 nt upstream of the AG-3’ acceptor site. The remaining, four NAT introns contained 5'-CU/AC-3’ splice site dinucleotides and are complementary to the introns found in the corresponding sense transcript. Putative branch point sites were found in all of these spliced NAT introns with non-canonical splice junctions.

### Antisense characterization in *U. maydis* and *U. hordei*

ESTs representing NATs in a given cDNA library indicated NAT presence within the cell type used to create the cDNA library. NAT presence at 96 loci was assessed by RT-PCR using RNA isolated from different cell types or nutritional conditions. The results are presented in Table S4. Oligo-d(T)_16_, water or strand-specific primers were used to prime reverse transcriptase reactions. This screen identified polyadenylated antisense transcripts consistent with their presence in cDNA libraries created from poly(A) enriched RNA. In this screen, oligo-d(T)_16_ could prime sense and/or antisense transcripts, during reverse transcription (RT). Due to the fact that the RT-PCR primers were designed within the overlapping sense–antisense transcript region, RT-PCR products resulting from oligo-d(T)_16_ primed cDNA have the potential to represent sense, antisense, or a mixture of the two transcripts. In the absence of an exogenous primer, RT reactions can be primed by RNA that has formed hairpin structures with itself, or by complementary endogenous RNAs (Sangar and Carroll, 1998), such as NATs. PCR products resulting from RT reactions lacking a primer (primed with water) were considered to be a result of false-priming. Finally, strand-specific primers were designed to specifically target the NAT during RT. As a whole, RT-PCR products using these different cDNA templates were used to assess the presence, or absence, of NATs in a given cell type.

**Fig. 1:**
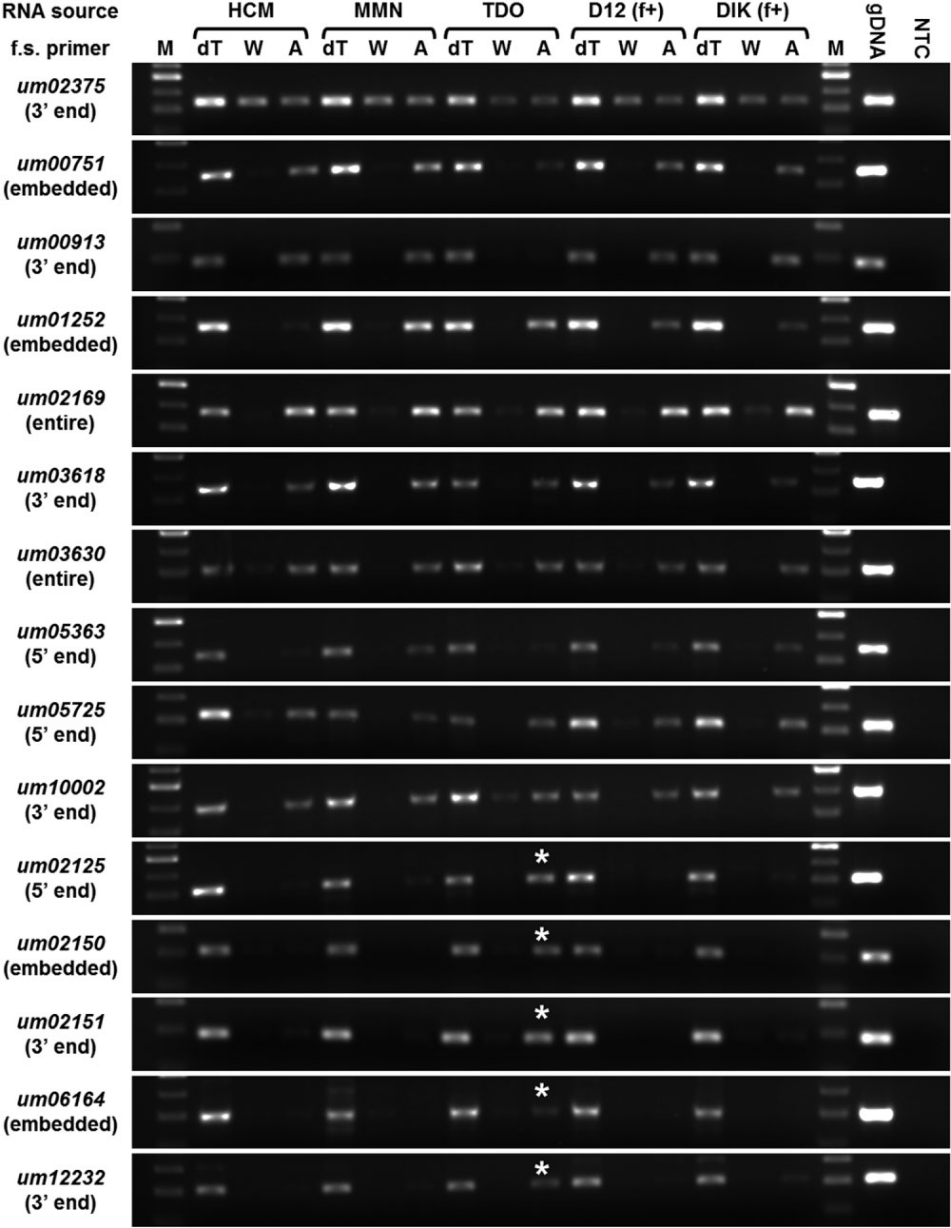
RT-PCR screen confirmed *in silico* analyses. The cellular origins of the RNA templates were: haploid cells grown in complete medium (HCM), haploid cells grown in nitrogen starvation medium (MMN), dormant teliospores (TDO), diploid filamentous cells (D12 f+) or dikaryotic filamentous mycelia (DIK f+). The primers used in first-strand synthesis (f.s.) were: oligo-(dT)_16_ (dT), water (W) or antisense-specific (A). The following were included as PCR controls: genomic DNA (gDNA) or no template (NTC). A DNA size marker (M) was also included. Dormant teliospore-specific increases in antisense transcript levels are indicated (*). ‘No reverse transcriptase’ controls were also performed and did not yield PCR products (data not shown).

Results for the 96 loci screened by RT-PCR are summarized in Table 2 and representative RT-PCRs are presented in Fig 1. There were 19 cases where background attributed to false-priming made the results inconclusive (e.g. *as-um02375*). Additionally, 11 NAT screens were difficult to interpret because of high background, but, in these, a NAT was detected in at least one cell type or nutritional condition. Only one NAT identified by EST analysis was not detected in the RT-PCR screen using 35 cycles, in any of the cell types or nutritional conditions (*as-um01439*). For the remaining 65 loci, NAT detection generally fell into three categories: (i) NATs detected at similar levels in all cell types (e.g. *as-um02169*), (ii) NATs detected at different levels in distinct cell types or nutritional conditions (e.g. *as-um01252*), or (iii) NATs with elevated levels in a specific cell type or nutritional condition and not detected in other cell types (e.g. *as-um02151*). The largest subset of NATs with elevated transcript levels in a specific cell type or nutritional condition were those detected in the dormant teliospore (Fig. 1, Table S4).

**Table 2:** Summary of RT-PCR screen confirming *in silico* analysis

RT-PCR remark^a^	Number of loci
NAT detected in all cell types and nutritional conditions	51
NAT enriched in TDO	6
NAT not detected in TDO	3
NAT enriched in MMN	2
NAT not detected in MMN	1
NAT not detected in any cell type or nutritional condition	1
NAT enriched in HCM, MMN and DIK (not detected in diploid cell types)	1
NAT enriched in HCM, MMN and TDO (not detected in filamentous cells)	1
High background (but NAT detected in at least one cell type or nutritional condition)	11
Inconclusive	19

**a.** The cellular origins of the RT-PCR templates were: haploid cells grown in complete medium (HCM), haploid cells grown in nitrogen starvation medium (MMN), dormant teliospores (TDO), diploid filamentous cells (D12) or dikaryotic filamentous mycelia (DIK).

NAT, natural antisense transcript.

**Fig. 2:**
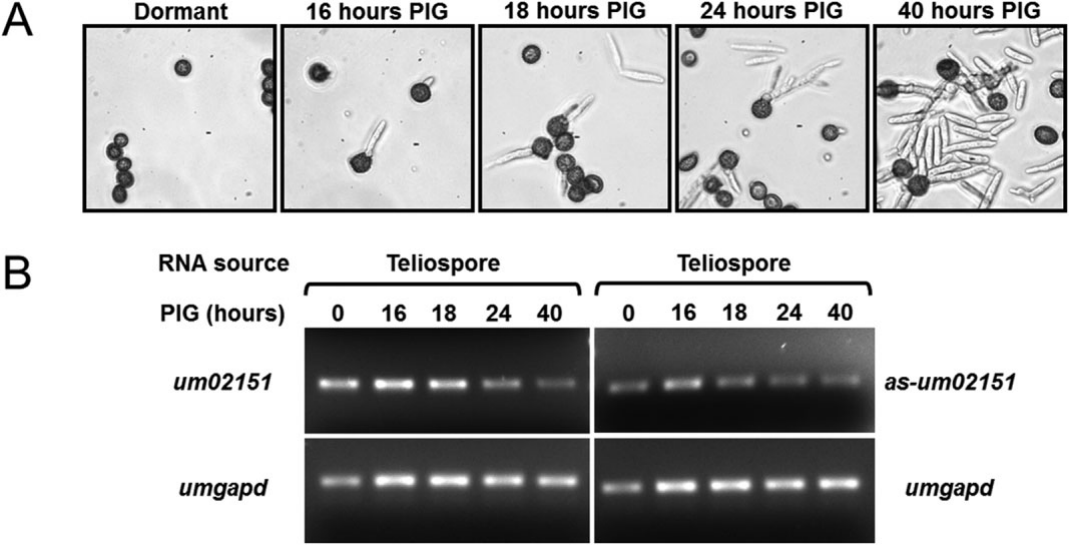
*um02151* sense and antisense (*as-um02151*) transcript levels decrease during teliospore germination. A. Representative images of teliospores at different time points post induction of germination (PIG). Dormant teliospores or germinating teliospores were observed microscopically (400× magnification). B. RT-PCR revealed an apparent decrease in the levels of *um02151* sense and antisense (*as-um02151*) transcripts as teliospores germinate. Equivalent concentrations of RNA were used as template for reverse transcriptase reactions in all lanes. Transcript-specific primers were used to generate cDNA for *um02151* or *as-um02151* and an internal *umgapd*-specific primer was used to assess transcript levels of the *gapd* ‘housekeeping’ gene.

The level of *as-um02151* was high in the dormant teliospore and undetected in other *U. maydis* cell types. RT-PCR was used to investigate the change in *um02151* transcript and *as-um02151* levels during teliospore germination. RNA was isolated from teliospores at four time points post induction of germination (PIG; Fig. 2A). Under the conditions used, teliospore germination was asynchronous and not all teliospores germinated. Elongating metabasidia were visible at 16 h PIG, and basidiospores and haploids were observed in increasing numbers at 18–40 h PIG. However, apparently dormant teliospores were detected at all time points (Fig. 2A). Transcript levels for *um02151* and *as-um02151* decrease during germination at a similar rate (Fig. 2B).

**Fig. 3:**
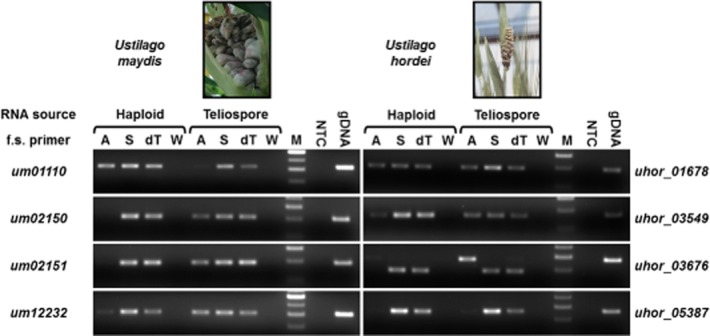
RT-PCR indicated the conservation of antisense expression between two *Ustilago* species. *U. maydis* (left) and *U. hordei* (right) infect corn and barley, respectively, causing the formation of tumours. RNA was isolated from haploid cells and teliospores, of *U. maydis* and *U. hordei*. The primers used in first-strand (f.s.) synthesis were: antisense-specific (A), sense-specific (S), oligo-(dT)_16_ (dT) or water (W). The following were included as PCR controls: genomic DNA (gDNA) and no template (NTC). A DNA marker (M) was run on the gels. ‘No reverse transcriptase’ controls were also performed and did not yield PCR products (data not shown).

Expression in response to an external stimulus or in a cell type-specific manner suggested a functional role for *U. maydis* NATs. The number of NATs specifically expressed in the dormant teliospore suggested that they may have a role in this cell type. The thick-walled teliospore is not unique to *U. maydis*; its formation is observed in the other smuts as well as the rust fungi. RT-PCR was used to examine whether *U. maydis* and a closely related smut, *U. hordei* (the causal agent of covered smut of barley), express similar NATs. Six loci were examined for the presence of *U. maydis* or *U. hordei* NATs in haploid cells or teliospores. At five of these loci, *U. maydis* teliospores exhibited enriched levels of NATs (*as-um02125*, *as-um02150*, *as-um02151*, *as-um06164* and *as-um12232*). The final locus, *um01110* (encoding a putative serine-protein kinase atr) was selected based on the observation that antisense transcript overexpression of a putative *um01110* orthologue in *S. cerevisiae* led to inhibition of cell growth (Nasr *et al*., 1994; 1995). RT-PCR results for select loci are presented in Fig. 3. Orthologous *U. hordei* NATs to *as-um06164* (data not shown) and *as-um12232* were not detected. However, in haploid cells and teliospores, orthologous *U. hordei* NATs were detected for *as-um01110*, *as-um02125* (data not shown) and *as-um02150*. A *U. hordei* NAT (*as-uhor_03676*), orthologous to *as-um02151*, was detected with enriched levels in the teliospore. In this instance, the PCR amplification product for *as-uhor_03676* was a different size than the amplicon for the *uhor_03676* sense strand using teliospore cDNA as template. The amplicon for *as-uhor_03676* was the same size as the PCR amplification using genomic DNA as template, suggesting the intron was not excised in the antisense transcript. The expression of orthologous NATs in a similar teliospore-specific manner implies a functional role for this antisense transcript.

**Fig. 4:**
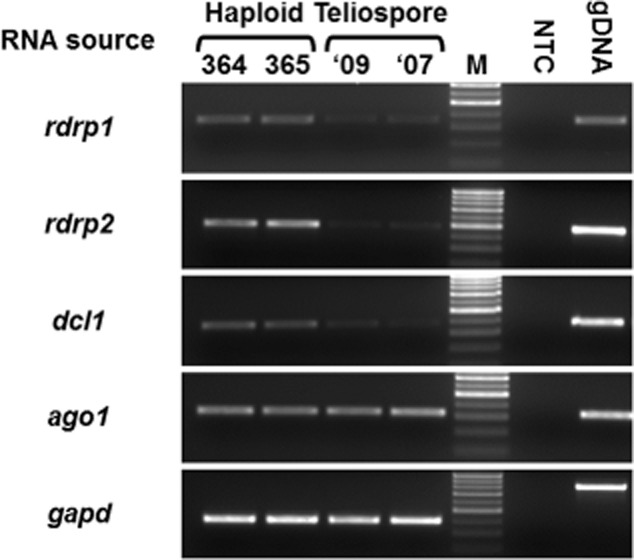
RT-PCR indicated that the transcript levels of key *U. hordei* RNAi pathway genes are reduced in dormant teliospores. RNA was isolated from *U. hordei* haploid strains *Uh364* (364) or *Uh365* (365), and mixtures of *U. hordei* teliospores collected from field samples across Manitoba and eastern Saskatchewan in 2007 (‘07) or 2009 (‘09). The following were included as PCR controls: genomic DNA (gDNA) or no template (NTC). A DNA marker (M) was run on the gels. ‘No reverse transcriptase’ controls were also performed and did not yield PCR products (data not shown).

In considering a possible functional role for the orthologue to *as-um02151* in *U. hordei* it is relevant to consider the fate of possible dsRNAs produced. If the RNAi machinery is present in the teliospore, these dsRNAs may have a different fate in *U. hordei* than in *U. maydis*. Therefore RT-PCR was used to determine if genes for key proteins in the RNAi pathway are expressed in the *U. hordei* teliospore. These screens indicated that transcript levels for two RNA-dependent RNA polymerases (*rdrp1* and *rdrp2*), and dicer (*dcl1*) are reduced in dormant teliospores relative to metabolically active haploid cells (Fig. 4). This suggests that RNAi function in the *U. hordei* teliospores is reduced or non-existent. This allows the possibility that dsRNAs in *U. hordei* and *U. maydis* could have similar fates.

**Fig. 5:**
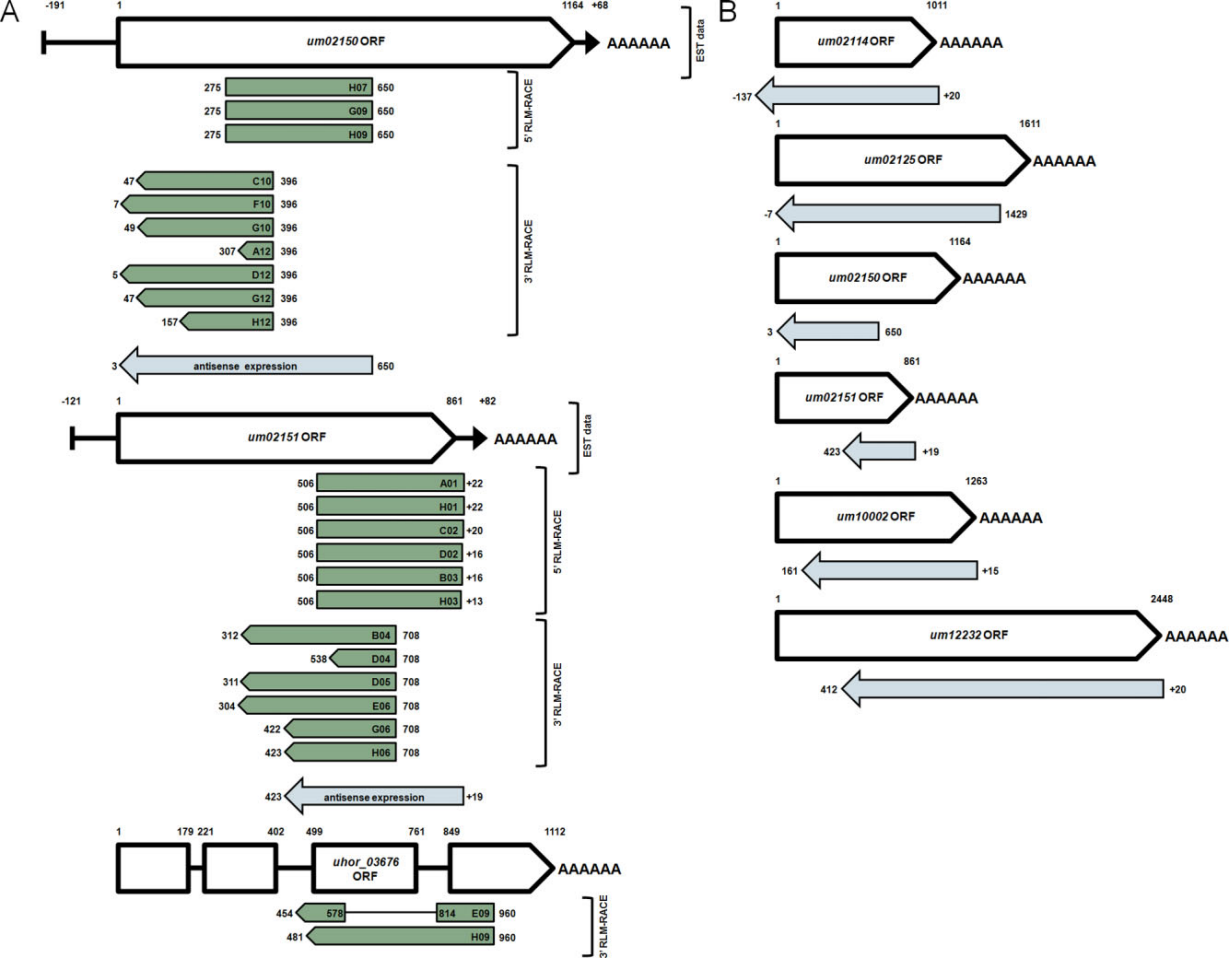
Schematic representation of antisense transcripts. A. Antisense transcript termini identified by RNA ligase mediated rapid amplification of cDNA ends (RLM-RACE). Sense strand open reading frames (ORFs) are represented by pentagons, with boxes separated by lines representing exons and introns respectively. The poly(A) tails are indicated by ‘AAAAAA’. *Ustilago maydis* EST data were used to estimate the lengths of sense strand 5’ untranslated regions (UTRs, vertical lines) and 3’ UTRs (arrows). No EST data were available for *Ustilago hordei*; therefore, putative *U. hordei* UTRs are not indicated. Sequences for 5’ or 3’ RLM-RACE products are represented by green boxes or green pentagons respectively. Clone identifiers are included with each RLM-RACE sequence. Numbers represent sequence position relative to the current ORF annotation. The region inserted into the autonomously replicating vector pCM768, expressed as an antisense transcript in haploid cells, is represented by blue arrows. B. Antisense transcripts expressed in *U. maydis* haploid cells. Six *U. maydis* antisense transcript expression vectors were constructed by inserting the regions depicted by the blue arrows into the autonomously replicating vector pCM768. Note that these regions were the annotated full-length NATs and that they have different regions of sense ORF overlap.

5’ and 3’ RACE was performed to map the ends of specific *U. maydis* NATs (*as-um02150* and *as-um02151*), as well as to annotate the *U. hordei as-uhor_03676* (Fig. 5A). Multiple 5’ and 3’ sequences were obtained representing the *U. maydis* NATs. Inspection of the 5’ sequences revealed very little variation (0 nt and 9 nt) for the start of transcription of *as-um02150* and *as-um02151*. The 3’ RACE sequences uncovered multiple polyadenylation sites for both antisense transcripts (Fig. 5A). For *U. hordei as-uhor_03676*, two sequences from 3’ RACE were obtained. Two different NATs at the *uhor_03676* locus were detected: one with an intron removed (clone E09), and the other with the intron retained (clone H09; Fig. 5A). Comparison of the sequence from clone E09 to the genome revealed 5'-GU/AG-3’ splice site dinucleotides, and a putative CUAAC branch point site 34 nt upstream of the AG-3’ acceptor site. Additionally, both NATs at the *uhor_03676* locus span intron splice junctions for the complementary sense transcript. The presence and expression pattern of antisense in these two smut fungi, regardless of the different structure in the sense transcripts (*U. hordei* with an intron and *U. maydis* without), adds support to a conserved role for these NATs, but may suggest distinct mechanisms of action.

Overall, the regions originally identified by sequencing clones from the teliospore cDNA library (depicted by the blue arrows in Fig. 5A) are similar to the combined results obtained by performing 5’ and 3’ RACE. Therefore, to create antisense transcript expression vectors, the antisense transcripts identified from the cDNA libraries were believed to be representative of the NAT population at a locus, and these sequences were inserted into the autonomously replicating vector pCM768 (Fig. 5B).

### Functional investigation of antisense in *U. maydis*

To investigate the function of *U. maydis* NATs, a 458 nt region of the genome corresponding to *as-um02151* was inserted into expression vector pCM768, and *U. maydis* haploid cells were transformed with the recombinant vector, FB1[pCMas-um02151]. *as-um02151* is detected by RT-PCR only in the teliospore (Fig. 1); therefore, *as-um02151* expression in haploid cells could be used to assess the effect of *as-um02151* on *um02151* transcript levels relative to the haploid cell transformed with an empty expression vector (FB1[pCM768]) which does not have antisense transcripts. Several independent transformants were isolated for FB1[pCMas-um02151]) and for FB1[pCM768]). RT-PCR confirmed *as-um02151* expression in FB1[pCMas-um02151] cells (Fig. S1). Additionally, RT-PCR revealed elevated *um02151* transcript levels in haploid cells transformed with vector expressing *as-um02151* (FB1[pCMas-um02151]), compared with haploid cells transformed with empty-vector, FB1[pCM768] (Fig. S1). Given this phenotype, more NATs were selected for expression studies.

pCM768 was used to drive the expression of five additional antisense transcripts in haploid cells. Additional antisense transcripts were chosen for analysis based on shared characteristics with *as-um02151*. These NATs overlap the 3’ end of the sense transcript, or showed elevated transcript levels in the teliospore, relative to other cell types or nutritional conditions (Table 3). RT-PCR was used to assess the sense transcript levels in vector transformed haploid strains expressing antisense, relative to empty-vector transformed haploid strains. These results are summarized in Table 3. In four cases, expression of antisense increased complementary sense transcript levels in haploid transformants, relative to control strains.

**Table 3:** Antisense expression summary

Sense transcript accession^a^	Type of NAT/ORF overlap	Length of NAT/ORF overlap (nt)	Antisense transcript expression^b^	Biological replicates (*n*)	Level of sense transcript^c^
*um02114*	Entire ORF	1011	Teliospore	5	No observed effect
*um02125*	5’	1429	Teliospore	8	No observed effect
*um02150*	Embedded	649	Teliospore	6	Increased
*um02151*	3’	439	Teliospore	8	Increased
*um10002*	3’	1101	All cell types	7	Increased
*um12232*	3’	2037	Teliospore	8	Increased

**a.** Accession according to MUMDB (http://mips.helmholtz-muenchen.de/genre/proj/ustilago/).

**b.** Antisense transcripts detected in specific cell types by RT-PCR.

**c.** Sense transcript levels in vector transformed haploid cells expressing antisense were estimated, relative to those observed in empty-vector transformed haploid cells, by RT-PCR.

NAT, natural antisense transcript; ORF, open reading frame; nr, non-redundant; nt, nucleotides.

For downstream functional investigations, emphasis was placed on studying the haploid cells transformed with vector expressing the *as-um02151* transcript. To gain an accurate measure of the effect of *as-um02151* transcript expression on *um02151* transcript levels using quantitative PCR (qPCR), amplification of false-primed cDNA needed to be mitigated. In a conventional strand-specific RT-PCR, the amplified PCR product can be a mixture of products from the primed and false-primed cDNA (Sangar and Carroll, 1998). To mitigate this potential problem, a ‘tagged’ *um02151* transcript-specific primer was used to prime the RT reaction. This primer contained a 5’ end composed of nucleotides not complementary to the *U. maydis* genome. The ‘tag’ sequence was incorporated into the cDNA which was used as template for PCR. During PCR, a *um02151* forward primer was used in conjunction with a reverse primer, specific for the tag sequence. This combination eliminated false priming, as measured by RT-PCR (Fig. S2A) and by TaqMan quantitative PCR (Fig. S2B).

**Fig. 6:**
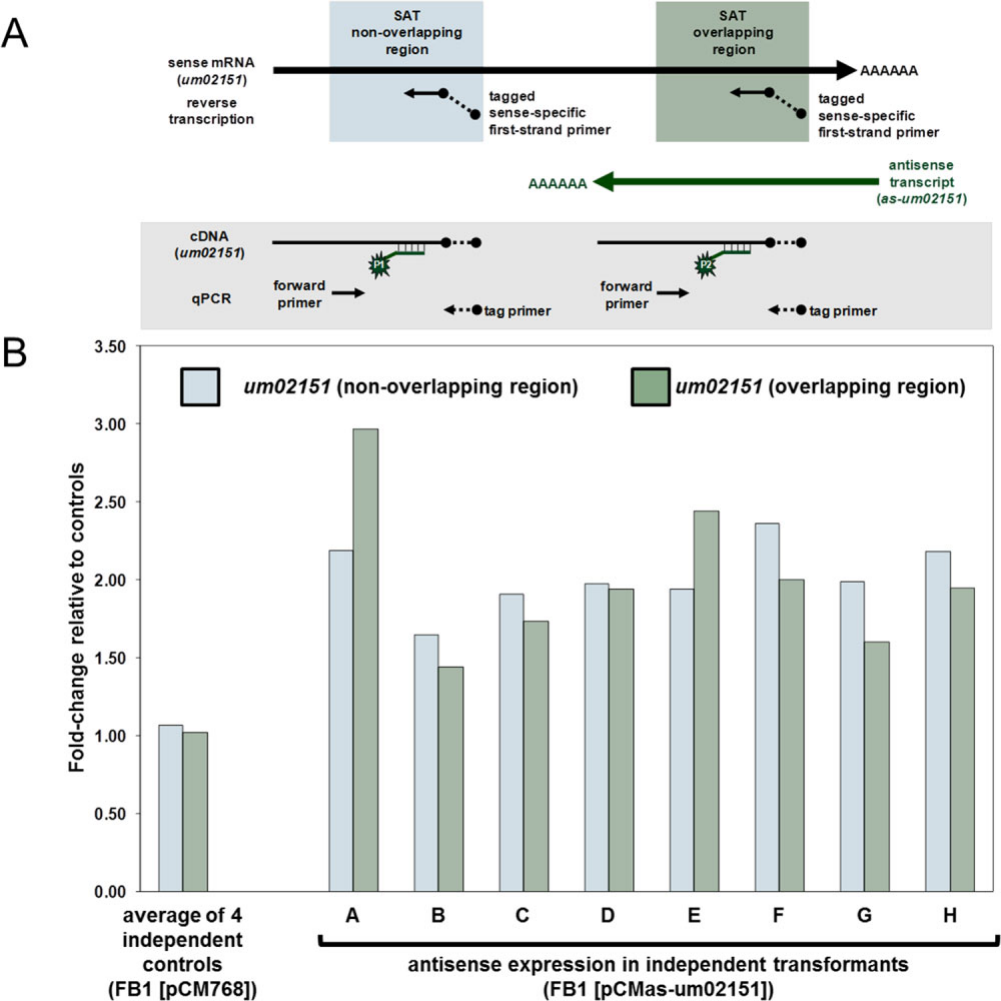
Expression of *as-um02151* increased *um02151* transcript levels in haploid cells. A. Schematic representation of tagged TaqMan quantitative PCR (qPCR). Tagged *um02151*-specific primers were used to generate cDNA for the non-overlapping sense–antisense transcript (SAT) region (blue box) or the overlapping SAT region (green box). The *um02151*-specific primers contain a 5’ tag (dashed line) which incorporates a sequence not complementary to the *U. maydis* genome into the cDNA. During TaqMan qPCR, the *um02151*-specific primer, tag-specific primer and *um02151*-specific probe (P1 or P2) contribute to a targeted quantification of the non-overlapping or overlapping portions of the *um02151* transcript. B. TaqMan qPCR comparative CT results. Using the formula 2^ΔΔ^^CT^, changes in *um02151* expression in eight independent vector transformed haploid strains expressing *as-um02151* (FB1[pCMas-um02151]) were analysed, relative to an average of four independent empty-vector transformed strains (FB1[pCM768]). For each sample, internal *umgapd* levels were used as a reference transcript.

TaqMan qPCR was performed to measure *um02151* transcript levels in vector transformed haploid strains expressing *as-um02151* (FB1[pCMas-um02151]) and empty-vector transformed strains (FB1[pCM768]). In separate qPCRs, the non-overlapping (blue box) or the overlapping *um02151/as-um02151* regions (green box) were amplified and detected using specific primer/probe combinations for the *um02151* transcript (Fig. 6A). Internal *umgapd* transcript levels were used as a reference to normalize *um02151* transcript levels in each sample. Using the formula 2^ΔΔCT^, *um02151* transcript levels were calculated for the independent FB1[pCMas-um02151] and FB1[pCM768] transformants, relative to average CT values obtained from the four FB1[pCM768] controls. While there was some variability, the average *um02151* transcript levels for the non-overlapping or overlapping regions were twofold higher in *as-um02151* expression strains (Fig. 6B).

**Fig. 7:**
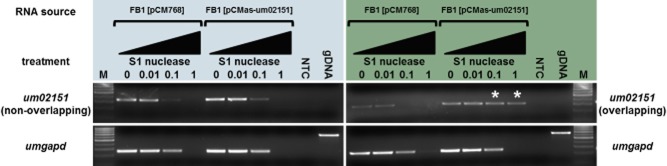
S1 nuclease digestion, followed by RT-PCR, confirmed that expression of *as-um02151* in haploid cells leads to double-stranded RNA formation. The origins of the RNA templates were: empty-vector transformed haploid cells (FB1[pCM768]), or vector transformed haploid cells expressing *as-um02151* (FB1[pCMas-um02151]). Equivalent concentrations of RNA were digested with increasing concentrations (U μl^−1^, final volume) of S1 nuclease. Tagged *um02151*-specific primers were used to generate cDNA for the non-overlapping sense–antisense region or the overlapping sense–antisense region. As a control, in all reverse transcriptase reactions, an internal *umgapd*-specific primer was also included to assess *gapd* transcript levels. The following were included as PCR controls: genomic DNA (gDNA) or no template (NTC). A DNA marker (M) was also included. Increased resistance to S1 nuclease digestion is indicated (*).

Given that expressing *as-um02151* in haploid cells led to increased levels of the corresponding *um02151* transcript, the potential for *um02151/as-um02151* double-stranded RNA (dsRNA) formation was investigated. RNA isolated from vector transformed haploid strains expressing *as-um02151* (FB1[pCMas-um02151]) and empty-vector transformed strains (FB1[pCM768]) was digested with increasing concentrations of S1 nuclease. For each S1 nuclease treated sample, separate RT reactions were primed with tagged *um02151*-specific primers, directed against the non-overlapping or overlapping *um02151/as-um02151* regions. RT-PCR targeting the *um02151/as-um02151* non-overlapping region showed susceptibility to S1 nuclease digestion for all treatments and samples. For the *um02151/as-um02151* overlapping region, resistance to S1 nuclease digestion was observed for FB1[pCMas-um02151] RNA compared with FB1[pCM768] RNA digested with 0.1 and 1.0 U μl^−1^ S1 nuclease. *umgapd* transcripts were susceptible to S1 nuclease in all experiments (Fig. 7). These results indicate the presence of an S1 nuclease-resistant transcript region only in the presence of antisense, and this suggests that *as-um02151* expression results in double-stranded (ds) *um02151/as-um02151* RNA formation.

**Fig. 8:**
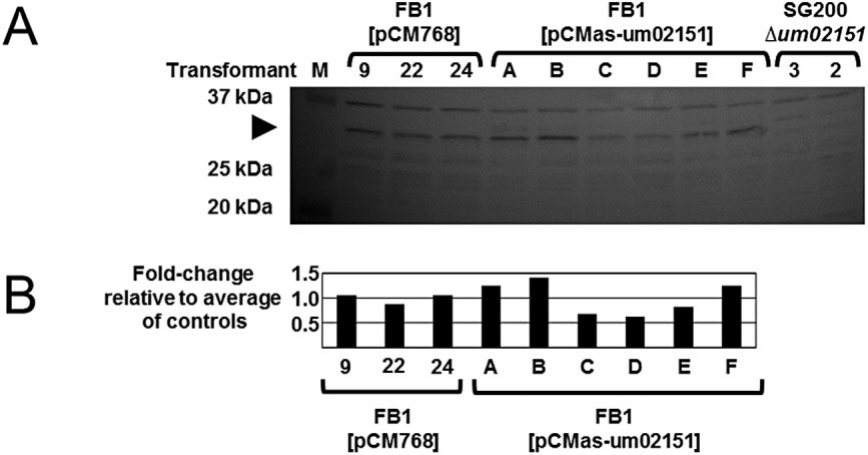
Expression of *as-um02151* did not alter Um02151 (protein) levels in haploid cells. A. Detection of Um02151 using Western blot. Total protein was extracted from three independent empty-vector transformed strains (FB1[pCM768]), six independent vector transformed haploid strains expressing *as-um02151* (FB1[pCMas-um02151]), and two independent *um02151* deletion strains (SG200Δ*um02151*). Equivalent concentrations of total protein were loaded into each lane, and separated by SDS-PAGE. The Western blot was developed with anti-Um02151 antibodies. A molecular weight marker (M) is included as a reference. The predicted band representing Um02151 (arrowhead) is present in FB1[pCM768] and FB1[pCMas-um02151], but absent in SG200Δ*um02151*. B. Um02151 levels approximated from Western blot. An image of the Western blot was taken using the Geliance 600 Imaging System (Perkin Elmer). To estimate Um02151 levels, the pixel volume for each sample was determined using GeneTools software (Perkin Elmer). Two separate Western blots contributed to the calculation of an average pixel volume, and the approximate level of Um02151 was recorded for each sample.

Given the expression of *as-um02151* in haploid cells led to increased levels of *um02151* transcript and ds *um02151/as-um02151* RNA formation, the impact of *as-um02151* expression on Um02151 (protein) levels was determined. Total protein was isolated from FB1[pCM768] and FB1[pCMas-um02151] strains and quantified by Bradford assay. Following electrophoretic separation of equivalent amount of total protein from all isolates, Western blotting was used to estimate protein levels. *um02151* encodes a putative protein related to 2,5-diketo-d-gluconic acid reductase with a predicted molecular weight of 32 kDa. Following Western blotting, the predicted band representing Um02151 was identified using the molecular weight ladder as a reference (in-between 25 kDa and 37 kDa), combined with the absence of a band in total protein isolated from *um02151* deletion strains (Fig. 8A). The relative pixel volumes obtained from two Western blots were averaged and plotted (Fig. 8B). These values were consistent with the band intensities observed on the Western blot. As a whole, Um02151 levels in FB1[pCMas-um02151] range from 0.66 to 1.41 times the levels found for the average of the FB1[pCM768] control strains (Fig. 8B). These results indicated that while expression of *as-um02151* in haploid cells led to an increased level of *um02151* transcript there was not a detectable increase in Um02151 protein levels. This suggests that the increased fraction of *um02151* sense transcript, detected by RT-qPCR, was not translated, likely because it was bound with the antisense transcript.

**Fig. 9:**
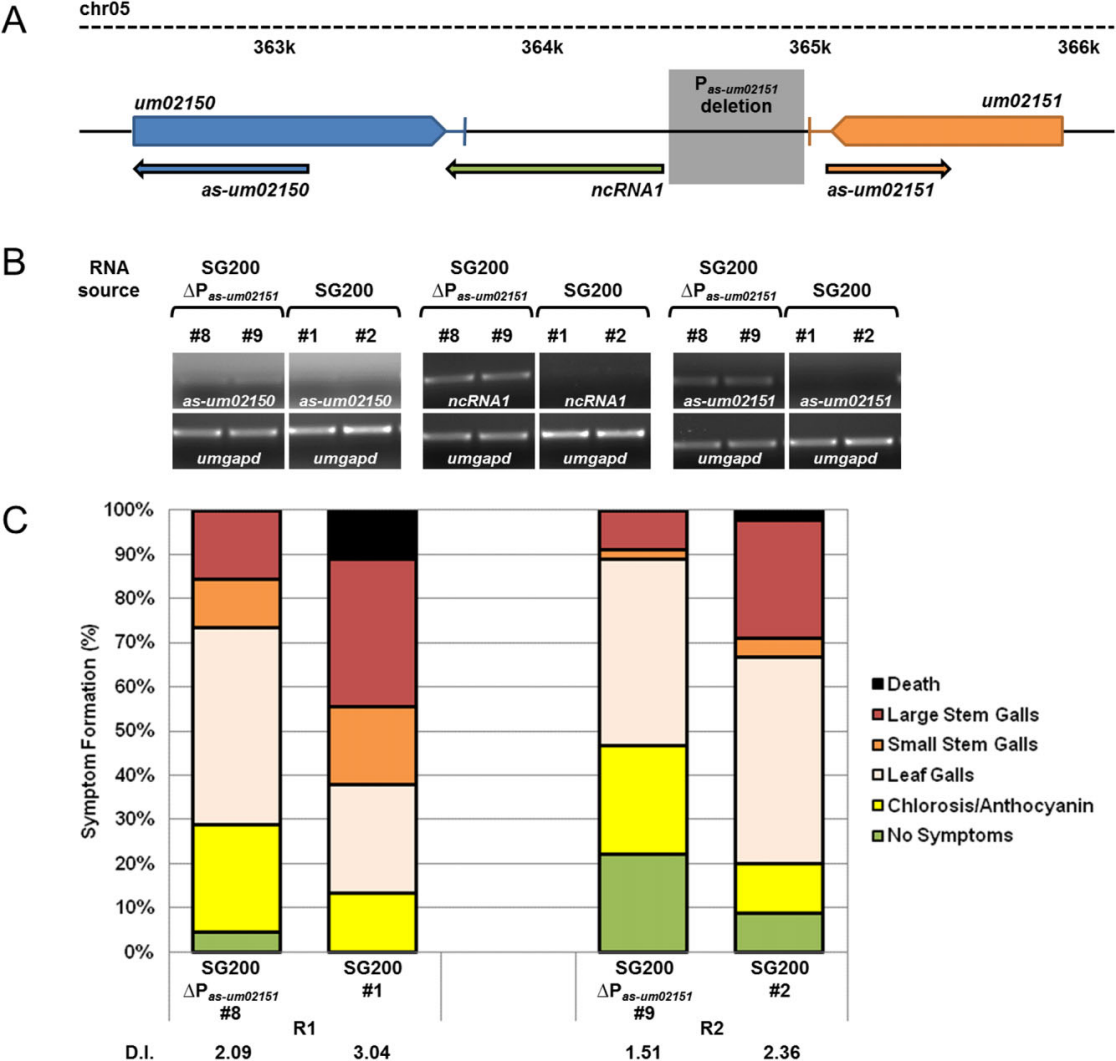
Deletion of P*_as_*_*-*_*_um02151_* attenuates pathogenicity. A. Schematic representation of the *um02150-um02151* locus. Annotated ORFs are represented by pentagons, putative 3’ UTRs by blue and orange vertical lines, intergenic regions by solid black lines and antisense transcripts by arrows. The region removed from SG200ΔP*_as_*_*-*_*_um02151_* strains is highlighted in grey. B. RT-PCR examining the effect of ΔP*_as_*_*-*_*_um02151_* on select transcript levels in haploid cells. RNA was isolated from independent SG200ΔP*_as_*_*-*_*_um02151_* transformants (#8 and #9) and independent SG200 controls (#1 and #2). Transcript-specific primers were used to generate cDNA for *as-um02150*, *ncRNA1* or *as-um02151*, and the housekeeping gene *umgapd*. ‘No reverse transcriptase’ and ‘no template’ controls were also performed (data not shown). C. Reduced pathogenicity of *U. maydis* SG200ΔP*_as_*_*-*_*_um02151_* strains. Infection with independent SG200ΔP*_as_*_*-*_*_um02151_* strains (#8 and #9) and their corresponding SG200 controls (#1 and #2) were performed in parallel. Seven-day-old maize seedlings (*n* = 45) were infected and the symptoms were scored 14 DPI. Separate infection assays (R1 and R2 respectively) differed slightly in the manifestation of disease symptoms. Bars represent the percentage of infected plants and the disease symptoms are depicted in the legend. The disease index (D.I.) for each strain is shown below the bar graph. Note that this experiment was performed in parallel with the pathogenesis assay reported in Morrison *et al*. (2012); therefore, the symptoms reported for the SG200 controls are the same.

Expression of *as-um02151* in haploid cells normally devoid of this NAT led to increased *um02151* sense levels, double-stranded *um02151/as-um02151* RNA formation, and unchanged Um02151 protein levels. Analysis of fully sequenced cDNAs (Doyle *et al*., 2011) enabled deletion of a region upstream of *as-um02151* (P*_as-um02151_*) in a solo-pathogenic *U. maydis* strain (SG200ΔP*_as-um02151_*) without removing the 3’ UTR for *um02151*, or deleting *ncRNA1*, a non-coding RNA previously described by Morrison *et al*. (2012) that is antisense to the 3’ UTR of *um02150* (Fig. 9A). RT-PCR results indicated that levels of *as-um02151*, as well as the adjacent antisense transcripts *as-um02150* and *ncRNA1*, were elevated, relative to SG200 in the *U. maydis* strain with this region deleted (SG200ΔP*_as-um02151_*; Fig. 9B). The transcript levels of the complementary *um02150* and *um02151* mRNAs at these loci were not altered by this deletion.

To investigate the impact of this deletion on antisense and mRNA transcript levels in the teliospores, ears of corn were injected with SG200ΔP*_as-um02151_* and SG200 cultures. However, ears of corn infected with SG200ΔP*_as-um02151_* failed to produce tumours or teliospores, while the controls, infected ears with SG200, produced tumours and teliospores normally (data not shown). To further investigate the impact of this deletion on pathogenesis, two independent seedling assays were carried out, comparing SG200ΔP*_as-um02151_* to SG200 infection. The assays were scored 14 days post infection (DPI) and they indicated that deletion of P*_as-um02151_* led to decreased virulence, relative to wild-type infections (Fig. 9C). This investigation failed to provide information on transcript levels in the teliospores; however, it indicated that altering NAT expression at these loci reduced the ability of *U. maydis* to infect corn. These data also suggested that the interpretation of results from gene deletion in *U. maydis* must include analysis of potential impacts on NAT expression from the targeted locus.

## Discussion

Annotation of the *U. maydis* genome established this fungus as the model for basidiomycete biotrophic plant pathogenesis (Kämper *et al*., 2006). Analyses of *U. maydis* cDNA libraries confirmed and corrected gene models, and led to the identification of natural antisense transcripts (NATs) to 247 non-redundant ORFs (Ho *et al*., 2007; Doyle *et al*., 2011; Morrison *et al*., 2012). In the current study, cDNAs representing NATs were fully sequenced, enabling their annotation. This information facilitated the characterization of NAT presence in specific *U. maydis* and *U. hordei* cell types. It also provided a basis for the functional role of NATs to be investigated in this model fungal pathogen.

### Natural antisense transcript annotation

Generation of full-length cDNA sequences representing antisense transcripts provided the ability to align long sequences to the genome. This strengthened annotation, relative to RNA-seq data, and allowed the identification of NATs complimentary to 3.6% of the predicted *U. maydis* ORFs. Similar examinations of cDNA libraries for *Cryptococcus neoformans*, *S. cerevisiae* and *Aspergillus flavus* discovered NATs to 0.8%, 5.1% and 2.8% of their predicted ORFs respectively (Loftus *et al*., 2005; Miura *et al*., 2006; Smith *et al*., 2008). However, strand-specific RNA-seq analysis allowed the identification of NATs to 16.7% of the *S. cerevisiae* ORFs (Yassour *et al*., 2010) and to 32.8–85.2% of the ORFs in other fungi (Gowda *et al*., 2006; Ohm *et al*., 2010; Tuch *et al*., 2010; Bitton *et al*., 2011). Similarly, more comprehensive RNA-seq investigations of *U. maydis* are expected to increase the number of NATs identified; however, the current full-length sequences enabled thorough NAT annotation.

*Ustilago maydis* NATs range from 149 nt to 2867 nt in length (median length 803 nt; Tables 1 and S1). This is similar to the NAT lengths reported for *S. cerevisiae* and *Schizosaccharomyces pombe* (reviewed in Harrison *et al*., 2009; Chen and Neiman, 2011). In *S. cerevisiae*, there is no obvious correlation between NAT length and the mechanism by which the NAT regulates gene expression (see table 1 in Donaldson and Saville, 2012). The majority of *U. maydis* NATs were embedded within ORFs, or complementary to the 3’ end of ORFs (Table 1). This is comparable to the most common class of NATs found in *C. neoformans* (embedded; Loftus *et al*., 2005), *S. cerevisiae* (3’ end; David *et al*., 2006; Miura *et al*., 2006) and *A. flavus* (3’ end; Smith *et al*., 2008), but differs from that of *C. albicans* (entire ORF; Sellam *et al*., 2010). Examination of the experimentally characterized NATs in *S. cerevisiae* revealed that they function via transcriptional interference or chromatin remodelling, independent of the position of the NAT/ORF overlap (Donaldson and Saville, 2012). However, it is possible that the position of overlap correlates to other mechanisms by which NATs alter gene expression. These functions may also relate to the length of the NAT.

Twenty-nine *U. maydis* NATs are complementary to at least one sense transcript splice junction, nearly half of which overlapped the entire intron (Tables S1 and S3). In the nucleus, sense–antisense double-stranded RNA formation may facilitate the production of alternatively spliced mRNA by ‘masking’ splice junctions from the spliceosome (Faghihi and Wahlestedt, 2009). Experiments in human cells indicated that endogenous expression of antisense overlapping the 5’ donor site of *Zeb2* mRNA led to intron retention and increased levels of Zeb2 protein (Beltran *et al*., 2008). In *U. maydis,* intron retention accounted for the majority of alternative splice events (Ho *et al*., 2007); however, the extent to which NATs control intron retention remains to be determined.

Full-length sequencing of cDNAs indicated ∼ 5% of *U. maydis* NATs had introns that were spliced to create the mature transcript. In one case, alternatively spliced antisense transcripts were found (*as-um10027*; Table S1). Intron removal is not a unique feature to *U. maydis*, as introns were detected for ∼ 27% of the *A. flavus* NATs identified through cDNA analysis (Smith *et al*., 2008). The lower percentage of NATs with introns in *U. maydis* is consistent with the observation that *U. maydis* has a lower number of introns per sense transcript than *A. flavus* (see table S2 in Donaldson and Saville, 2012). With the exception of *S. cerevisiae* (mean length 256 nt), *U. maydis* has relatively large introns (mean length 170 nt) compared with other fungi (Ho *et al*., 2007). The introns annotated in *U. maydis* NATs are even larger; nearly double the length of those within *U. maydis* sense transcripts. This size difference may result from a lack of comprehensive *U. maydis* NAT identification, but the presence of 5'-GU/AG-3’ splice site dinucleotides, and CURAY or YYRAY branch point sites, suggested that NAT introns are structurally comparable to those found in mammals, other fungi and *U. maydis* sense transcripts (Burset *et al*., 2001; Kupfer *et al*., 2004; Ho *et al*., 2007). Interestingly, 5'-CU/AC-3’ splice site dinucleotides were identified in four NAT introns. ESTs with these splice site dinucleotides were removed from *A. flavus* NAT analysis (Smith *et al*., 2008), but perhaps this represents a rare, yet active, donor-acceptor pair. Further NAT characterization is required to confirm these splice junctions.

In total, 155 *U. maydis* NATs contained ORFs encoding proteins > 50 aa. The ORFs in *as01-um01110* and *as01-um03351* encode putative proteins that are similar to hypothetical proteins in other fungi (Table S2). A *S. cerevisiae* NAT, embedded within the *MDF1* ORF, encodes a protein (Adf1) that acts as a repressor of *MDF1* (Li *et al*., 2010), and 50 NATs in *A. flavus* were predicted to encode proteins (Smith *et al*., 2008), so fungal NATs may increase the protein coding potential of a genome. The putative ORF for *as01-um10078* encodes a protein with similarity to a *U. hordei* uncharacterized protein UHOR_03688 (Table S2). Comparison of *uhor_03688* to *as01-um10078*, and not *um10078*, revealed conserved synteny, suggesting *um10078* is a questionable ORF, and that the *as01-um10078* ORF is the correct annotation at this locus. In this case, annotating the putative antisense corrected the genome annotation. Two *U. maydis* NATs, *as01-um00047.2* and *as01-um06390*, overlap the ORF of the adjacent gene on the same strand, and one of the ORFs predicted for each NAT is the overlapped sense ORF, so the protein coding potential for these two antisense is unclear (Table S2). However, the NATs could influence expression of the ORF they are complementary to, as well as the divergently transcribed adjacent ORF, in a manner similar to the *S. cerevisiae GAL1-10* locus (Houseley *et al*., 2008). Excluding the NATs discussed above, the remaining 150 *U. maydis* NAT ORFs were used to predict encoded peptides and these were inspected for similarity to known proteins, and secretion signals. None of the predicted proteins encoded by NATs were similar to a protein of known function in the NCBI database. This pool of NATs may increase the protein coding potential for the *U. maydis* genome, or they may represent functional non-coding RNAs.

An intriguing possibility is that some *U. maydis* NATs encode effector proteins, or are non-coding NATs that alter effector protein expression. Broadly speaking, fungal effectors are small molecules or proteins that interact with the host, effecting virulence by interfering with plant defences (reviewed in Brefort *et al*., 2009; Ali and Bakkeren, 2011; Koeck *et al*., 2011). Recently, expression of the *U. maydis* effector Pit2 (93 aa), was identified as a requirement for tumour formation (Doehlemann *et al*., 2011a). *U. maydis* effector proteins typically have a secretion signal, are upregulated during *in planta* growth, and lack similarity to known proteins (reviewed in Doehlemann *et al*., 2011b). Predicted proteins for eight *U. maydis* NATs contain recognizable secretion signals. Similar to effectors in *Cladosporium fulvum* (reviewed in Stergiopoulos and de Wit, 2009), these NATs would encode relatively short processed peptides, with lengths ranging from 30 to 102 aa (Table S2). This suggested that the putative short peptides encoded by *U. maydis* NATs require further investigation. One of these, encoded by *as-um12291*, may be of particular interest because it is complementary to a gene within the *U. maydis* effector cluster 15-12. Deletion of cluster 15-12 led to reduced virulence in *U. maydis-*infected seedlings, and as annotated, the cluster encodes three proteins lacking recognizable secretion signals (Schirawski *et al*., 2010). The *as-um12291* encoded protein may add to the function of this cluster, or it may indicate an error in the original annotation. A further six *U. maydis* genes encoding effectors have complementary NATs, including an additional NAT, complementary to *um04936* in the cluster 15-12. Additionally, key effectors expressed during biotrophic growth, such as Cmu1 and Pit1, have antisense transcripts at their loci (Djamei *et al*., 2011; Doehlemann *et al*., 2011a). Together, these observations have three implications: (i) that the putative secreted peptide encoded by *as-um12291* plays a role in the phenotype observed by Schirawski *et al*. (2010), (ii) additional effectors lacking secretion signals may exist, some of which are encoded by *U. maydis* NATs, and (iii) NATs at effector loci may influence effector expression.

Overall, *U. maydis* NAT annotation revealed transcripts that have structural features similar to those of other fungi. They have similar lengths, some have introns and some potentially encode proteins. The protein encoding NATs include short transit peptide-containing proteins that may be effectors; therefore, these NATs could increase the protein coding capacity of the fungus, and may do so in a manner that influences pathogenesis. However, there are also a substantial number of NATs that do not encode proteins and may have a function in the regulation of gene expression. The levels of a subset of these NATs were assessed in a range of cell types, setting the stage for a targeted functional investigation.

### Antisense characterization in *U. maydis* and *U. hordei*

Strand-specific RT-PCR supported *in silico* NAT predictions and provided information regarding the presence of NATs in specific cell types or under distinct nutritional conditions (Tables 2 and S4, Fig. 1). The majority of NATs were detected at similar or varying levels in all cell types (Tables 2 and S4, Fig. 1). In this initial screen, sense transcript levels were not determined, so the ratio of sense to antisense transcript was not investigated. Constitutively expressed NATs were also detected in other fungi (Donaldson and Saville, 2012). The role of these NATs has not been well studied in any eukaryote and will be the subject of future investigations in *U. maydis*. The focus of the work presented here is a group of NATs expressed in a cell type-specific manner, notably those expressed preferentially in the teliospore.

A functional role for eukaryotic antisense transcripts is supported by tissue- or stimuli-specific expression (Beiter *et al*., 2009). Tissue-specific NAT expression has been noted for *S. commune*, *S. cerevisiae* and *S. pombe*; and in the yeasts there is NAT-mediated suppression of meiosis gene expression (Chen and Neiman, 2011; Donaldson and Saville, 2012). In *U. maydis*, six NATs had elevated levels in dormant teliospores (Tables 2 and S4, Fig. 1). Strand-specific RT-PCR results indicated that the levels of sense and antisense transcripts in teliospores were similar, and that the sense transcripts were also detected in haploid cells (data not shown). Therefore, NAT expression in the teliospore does not exclude sense transcript expression, as has been shown for many *S. cerevisiae* and *S. pombe* sense–antisense transcript pairs. Rather, the similar levels of sense and antisense transcripts suggested an interaction between the pairs that exists in the teliospores, but not the haploid cells.

Sense and antisense transcript levels decreased during germination for one of the six loci (*um02151*) with teliospore-specific antisense (Fig. 2B). Decreasing transcript levels have been detected during spore germination in a number of fungi. The mRNAs, ‘stored’ or ‘pre-packaged’ in the spores, may be utilized during dormancy, or they may be translated following the initiation of germination (Osherov and May, 2000; Brengues *et al*., 2002; Sacadura and Saville, 2003; Zahiri *et al*., 2005; Liu *et al*., 2007; Lamarre *et al*., 2008). Translation was detected in the presence of transcription inhibitors during spore germination in *Aspergillus nidulans* (Osherov and May, 2000), supporting the storage of some mRNAs for later translation. These previous studies were not designed to detect NATs, so it is unknown if there are NATs complementary to the stored mRNAs identified in the spores of other fungi. Functional Catalogue (FunCat) entries indicate that the six *U. maydis* genes with teliospore-specific antisense encode proteins active in metabolism, energy and transcription; yet, the teliospore is dormant. This suggested the encoded proteins are translated during germination when the teliospore is becoming metabolically active. The presence of stored sense–antisense transcript pairs in *U. maydis* teliospores would suggest that NATs may be involved in facilitating long-term storage of their complementary sense transcripts so that they are available for rapid translation once germination is initiated.

Teliospore formation is characteristic of the smut and rust fungi; therefore, it was of interest to investigate whether antisense expression was unique to *U. maydis* or present in other related smuts. Strand-specific RT-PCR detected four NATs conserved between *U. hordei* and *U. maydis*. One of these NATs, *as-uhor_03676*, is orthologous to *as-um02151*, and the transcript was preferentially detected in teliospores like the *U. maydis* counterpart (Fig. 3). The conservation of NATs between these smuts is noteworthy because they differ in their ability to perform RNA interference. *U. hordei* has RNAi machinery, but *U. maydis* does not (Laurie *et al*., 2008). Therefore, one might expect that sense–antisense transcripts detected in the same cell type have the potential to form dsRNA, and that if the ribonuclease III enzyme, Dicer, was present, this dsRNA would be cleaved. This suggested that dsRNA formed in these two smut fungi have different fates. However, restricting expression to a specific cell type seems to have eliminated this difference, since RT-PCR indicated that the transcript levels of RNAi genes *rdrp1*, *rdrp2* and *dcl1* are reduced in the *U. hordei* dormant teliospore (Fig. 4). *U. hordei* NATs expressed in the teliospore could form dsRNA under conditions where they would be ‘protected’ due to low levels of Dicer, and their function may be similar to the teliospore-specific NATs discovered in *U. maydis*. While it is possible that these NATs do not have a function, the data strongly suggested that: (i) *U. hordei* and *U. maydis* share a group of NATs that act in an RNAi-independent manner to influence gene expression in a similar or species-specific manner, and (ii) the loss of the RNAi machinery may have enabled new instances of NAT-mediated gene expression control to evolve in *U. maydis*, but not in the smut fungi that have this machinery.

Loss of the RNAi silencing machinery in *U. maydis* has been attributed to an efficient homologous recombination system (Holloman *et al*., 2008; Laurie *et al*., 2012), or enabling the acquisition of dsRNA killer viruses (Drinnenberg *et al*., (2011). The observations and proposals presented here, based on comparison of *U. maydis* and *U. hordei*, are consistent with this; however, they extend it to the possibility that some NATs function in ancient roles conserved among the smut fungi, and other NATs have evolved to take on distinct roles in the absence of constraint by RNAi. These evolved roles could lead to the fungi being better able to occupy specific ecological or host niches which may have provided a selective advantage.

### Functional investigation of antisense in *U. maydis*

RT-PCR results revealed that expression of *as-um02151* led to increased levels of the complementary *um02151* transcripts (Fig. S1). Five other *U. maydis* NATs were expressed in haploid cells. Three of these similarly led to an increased level of complementary mRNA (Table 3). Among these NATs, no universally common features were observed, which suggested that the interactions mediating their functions may be complex.

Detecting *um02151* transcript levels that were twofold higher in *as-um02151* expression strains relative to vector only control strains (Fig. 6B) was enabled by using a tagged *um02151*-specific primer during reverse transcription and a complementary primer during qPCR (Fig. S2; Gu *et al*., 2007; Plaskon *et al*., 2009). This result contrasted the situation in budding yeast, where NAT expression led to chromatin remodelling or transcriptional interference, which reduced the level of sense transcript (Harrison *et al*., 2009; Donaldson and Saville, 2012). There is one exception to this in *S. cerevisiae*, where naturally constitutive transcription of antisense at the *PHO5* locus enabled rapid initiation of *PHO5* transcription (increased sense transcript level) under phosphate limiting conditions (Uhler *et al*., 2007). The *U. maydis* experiment presented here involved NAT expression from an autonomously replicating plasmid, indicating the mechanism of sense transcript increase was distinct from that in budding yeast.

Transcript levels are a result of transcription and degradation. *as-um02151* RNA could increase the level of the complementary *um02151* mRNA by inhibiting degradation through the formation of double-stranded RNA (dsRNA). dsRNA formation in the nucleus has the potential to interfere with the mRNA–protein interactions required for splicing or transcript localization/export (reviewed in Lavorgna *et al*., 2004; Beiter *et al*., 2009; Faghihi and Wahlestedt, 2009). In mammals, cytoplasmic dsRNA formation has been directly linked to increased mRNA stability (Faghihi *et al*., 2008; Matsui *et al*., 2008). In *S. cerevisiae*, dsRNA formation led to the production of a truncated Kcs1 protein (Nishizawa *et al*., 2008). The expression of *as-um02151* in *U. maydis* haploid cells led to dsRNA formation (Fig. 7); however, the amount of *um02151* transcript present in the dsRNA hybrids was not quantified, nor was the level of *as-um02151* transcript derived from the plasmid. Therefore, any variation in these levels among *as-um02151* expressing strains was not detected. The increased level of *um02151* mRNA did not result in higher protein levels; however, this interpretation is limited by the lack of comparison to a reference protein that is expressed at constant levels in cells transformed with an empty vector and with the vector expressing the *as-um02151* transcript. Identifying such a reference protein would improve the ability to detect changes in protein levels resulting from *as-um02151* expression and would provide more definitive evidence for translation inhibition as a result of dsRNA formation. Acknowledging these limitations, the increased *um02151* transcript level, and the inability to detect a change in Um02151 protein level, or to detect truncated protein products (Fig. 8A), suggested that a fraction of the sense transcript was bound in dsRNA and not translated, while a separate fraction was unbound and translated. These two subpopulations represented more *um02151* transcript than is present in cells without NAT expression.

**Fig. 10:**
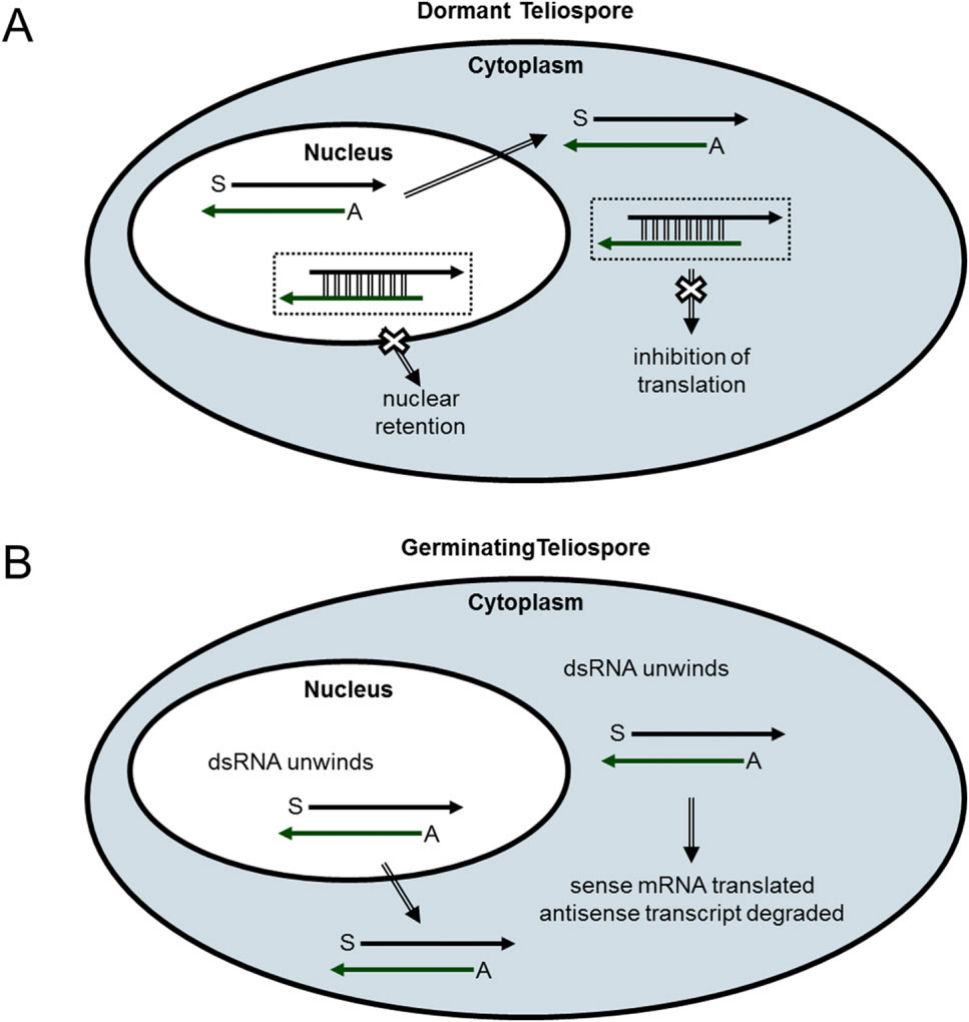
Proposed model illustrating a functional role for antisense transcripts expressed in *U. maydis* teliospores. A. Antisense transcripts facilitate dsRNA formation in dormant teliospores. Formation of dsRNA (double-lines) in the nucleus may prevent export of the sense transcript from the nucleus. Alternatively, dsRNA may form in the cytoplasm. In either case, mRNA (S, black arrow) bound by antisense RNA (A, green arrow) is inhibited from being translated. B. In response to external stimuli, dormant teliospores become metabolically active, germination is initiated and dsRNA is unwound. This process allows mRNAs to be translated and then degraded, while antisense RNAs are degraded.

It is interesting to consider the physiological response of *U. maydis* cells to these subpopulations of *um02151* transcripts. In metabolically active haploid cells, the presence of antisense transcripts resulted in dsRNA formation, translation inhibition and stabilization of the bound sense transcripts. This increased the steady-state mRNA levels; however, similar protein levels were produced. This suggested the haploid cells compensated for the bound mRNA by transcribing more mRNA. In the dormant teliospore, dsRNA formation and the reduction of translation may not require compensation because these cells have very low metabolic activity and would not require ongoing *um02151* translation. In this scenario most, or all, of the *um02151* mRNA would be bound as dsRNA and not be translated, but rather stored for translation upon germination. This was extrapolated to a model in which antisense transcripts stabilize mRNA in the dormant teliospore through dsRNA formation (Fig. 10). If this dsRNA is in the nucleus, it would lead to retention of the sense transcript, whereas, if it was in the cytoplasm, translation would be blocked (Fig. 10A). Upon germination, the dsRNA hybrid is unwound and the mRNA is translated then degraded, while the antisense transcript is degraded directly (Fig. 10B). The *U. maydis* genome contains genes encoding an estimated 35 RNA helicases (data not shown), one of these (Um05767) is related to ATP-dependant RNA helicases, and it has a dsRNA-binding domain. Transcripts of *um05767* were detected in the teliospore (data not shown). If the protein is also present, it would be inactive because of the low respiration rate in the teliospore (Caltrider and Gottlieb, 1963). Prior to germination, the increased metabolic activity would lead to translation and/or activation of Um05767, the separation of dsRNAs, including *um02151/as-um02151* dsRNA, and increased protein expression.

In an attempt to test the hypothesis that NAT expression in *U. maydis* teliospores stabilizes mRNAs for translation upon germination, the upstream region of *as-um02151* was deleted (Fig. 9A) in a solo-pathogenic haploid strain (SG200ΔP*_as-um02151_*). The results of this were similar to the deletion of *ncRNA1*, positioned just upstream of P*_as-um02151_* (Morrison *et al*., 2012). Both deletions led to increased transcript levels of *as-um02150* and *as-um02151,* which suggested the deleted regions have regulatory functions. The deletions did not affect the levels of *um02150* and *um02151* mRNA; however, these transcript levels were assessed through RT-PCR and this would not have allowed the detection of a small increase in *um02151* mRNA level due to binding with the low level of *as-um02151* transcript expressed. Deletion of the *as-um02151* upstream region also led to increased *ncRNA1* transcript levels (Fig. 9B; fig. 3 in Morrison *et al*., 2012). This suggested that the expression of *as-um02150*, *ncRNA1* and *as-um02151* are controlled by a repressor binding to a bidirectional regulatory region. The *ncRNA1* deletion results (Morrison *et al*., 2012) indicated that this repressor also has binding sites within the *ncRNA1* coding region. The presence of these binding sites means that *ncRNA1* deletion not only removes the RNA, but also reduces repressor binding. This led to an increase in *as-um02150* and *as-um02151* transcript levels. During the *U. maydis* life cycle, the proposed repressor would be expressed in haploid cells but not in teliospores where NAT expression was de-repressed. The timing of this de-repression was not investigated because these deletions altered pathogenesis. Ears of corn infected with the NAT regulatory region deletion strains failed to produce teliospores, and in seedling assays, they showed reduced virulence (Fig. 9C). This indicated that altering the expression of these NATs reduced virulence.

*Ustilago maydis* NATs may have multiple roles that influence virulence. Annotation indicated some NATs potentially encode effector proteins and others are complementary to genes known to be involved in pathogenesis. This suggested that some NATs are actually effector mRNAs and others have a broad role in controlling gene expression leading to normal pathogenesis. Further, the distribution of the NATs in the genome and conservation of complementary genes among the smuts, suggested that some roles of NATs in pathogenesis existed prior to their divergence, and others after smut divergence to new hosts. Since *U. maydis* lacks the RNAi machinery and RNAi mutants in the related *S. reilianum* retain normal pathogenesis (Schirawski *et al*., 2010) the NAT-mediated pathogenesis effects must act independently of RNAi pathways. The mechanisms by which NAT expression influences pathogenesis are the focus of ongoing research.

In this study, *U. maydis* NATs were annotated and the expression of select NATs in haploid cells led to increased levels of some complementary mRNAs. The function of one such NAT was further investigated and led to the development of a model in which *U. maydis* NATs preferentially expressed in the teliospore have roles in the protection of stored mRNAs during dormancy and the control of stored mRNA translation upon germination. Manipulating the expression of NATs identified a role for NATs in *U. maydis* pathogenesis. Future investigation of other identified NATs is expected to identify further novel roles for NATs in fungi.

## Experimental procedures

### Antisense transcript characterization

*Ustilago maydis* antisense transcripts were identified by Ho *et al*. (2007) and Morrison *et al*. (2012) through the analysis of 5’ ESTs represented in *U. maydis* cDNA libraries created from germinating (T11) and dormant (TDO) teliospores (Sacadura and Saville, 2003; Ho *et al*., 2007), haploid cells grown in complete medium (HCM), carbon starvation medium (MMC) or nitrogen starvation medium (MMN; Ho *et al*., 2007), forced diploids grown filamentously (D12; Nugent *et al*., 2004) or filamentous dikaryotic mycelia (DIK; Morrison *et al*., 2012).

Clones representing antisense transcripts were selected for 3’ sequencing. Plasmid DNA isolation followed the procedure outlined in Nugent *et al*. (2004). Sequencing from the 3’ end of the antisense transcript was performed using primers M13_R or dT_19_V (Table S5). Nucleotide sequences were determined using Big Dye Terminator v3.1 chemistry (Applied Biosystems) and the reaction products were analysed using an ABI PRISM 3730 DNA Analyser (Applied Biosystems).

New ESTs were edited to remove contaminating vector sequences as indicated in Morrison *et al*. (2012), except that all sequences were manually inspected for the presence of a poly(A) tail. The 5’ and 3’ ESTs representing antisense transcripts were aligned using blastn to the *U. maydis* chromosome assembly file ‘Umaydis_contigs.fas’ (last modified 8 September 2011) or the *U. maydis* open reading frame (ORF) file ‘Umaydis_valid_orf.fas’ (last modified 24 May 2011). These FASTA (.fas) files were downloaded from the Munich Information Centre for Protein Sequences (MIPS) *U. maydis* database (MUMDB; Mewes *et al*., 2008). All 5’ and 3’ ESTs from clones representing antisense transcripts were confirmed to contain the antisense strand of a MUMDB gene. Additionally, in aligning the pair of ESTs representing the 5’ and 3’ ends of an antisense transcript to the *U. maydis* genome, or *U. maydis* ORFs, characteristics of antisense transcripts were determined, including: the length of the antisense transcript, the type and length of overlap (with respect to the ORF), and those antisense transcripts which contain an intron. Antisense transcripts containing an intron were confirmed manually and the splice site nucleotides were recorded. Putative ORFs within full-length NATs were detected and used to predict their corresponding amino acid sequence using Geneious Pro v5.6.5 (Biomatters). The resulting putative proteins were used in blastp searches (conducted in October 2012) against the NCBI non-redundant protein database with an expect threshold of E < 1e-5. For each protein, only the best blastp hit was reported. Furthermore, putative proteins were inspected for N-terminal secretion signals using SignalP v4.0 (Petersen *et al*., 2011), TargetP v1.1 (Emanuelsson *et al*., 2000), and ProtComp v9.0 (Softberry). Only those proteins predicted to have extracellular location by all three programs were recorded.

### Plasmid and strain constructions

All procedures were carried out as suggested by the manufacturers, unless otherwise stated. Plasmids utilized in this study are listed in Table S6. The *U. maydis* expression vector pCM768 is a non-integrating vector that contains a *U. maydis* autonomously replicating sequence (ARS), a hygromycin B resistance (Hyg^R^) cassette, and the *U. maydis* glyceraldehyde-3-phosphate dehydrogenase promoter (P*_umgapd_*) which facilitates the expression of genes inserted in the multiple cloning site (Kojic and Holloman, 2000). To express antisense transcripts from an autonomously replicating vector, a region of the genome corresponding to an antisense transcript (identified through full-length cDNA analysis) was amplified by PCR using primers which introduce restriction endonuclease recognition sequences at their 5’ ends (Table S5). The PCR fragment and pCM768 were digested with restriction endonucleases and purified. Linearized pCM768 was treated with Antarctic Phosphatase (New England BioLabs) and ligated to the purified digested PCR fragment. The resulting *U. maydis* antisense transcript expression vector was transformed into Subcloning Efficiency DH5α Competent Cells (Invitrogen). Six *U. maydis* antisense transcript expression vectors were constructed using this approach (Table S6). The correct nucleotide sequence and orientation for the region of the genome encoding the antisense transcript was confirmed by sequencing. All sequencing reactions were performed using primer pgapd_79_F (Table S5). FB1 or FB2 protoplasts were transformed with the antisense expression vector, using a modification of the Yee (1998) protocol, previously described in Morrison *et al*. (2012). Genomic DNA was isolated from putative transformants using the protocol outlined by Hoffman and Winston (1987). Successful *U. maydis* transformants were confirmed using PCR. PCRs were performed to amplify a region of the Hyg^R^ cassette using primers pCM768_Hyg_F and pCM768_Hyg_R, or the P*_umgapd_*-driven antisense transcript, using the primer pgapd_79_F and the reverse primer used to clone the genomic region corresponding to the antisense transcript (Table S5). Furthermore, antisense transcript expression was confirmed via strand-specific semi-quantitative RT-PCR (see transcript expression analysis, below). The *U. maydis* FB1- and FB2-derived strains created for antisense transcript expression analyses are listed in Table 4. In each case, several strains were identified.

**Table 4:** Strains used in this study

Strain	Relevant genotype	Source
***Ustilago maydis***		
518	*a2 b2*	Holliday (1961)
521^a^	*a1 b1*	Holliday (1961)
d132	*a1/a2 b1/b2*	Kronstad and Leong (1989)
FB1	*a1 b1*	Banuett and Herskowitz (1989)
FB2	*a2 b2*	Banuett and Herskowitz (1989)
FBD12	*a1/a2 b1/b2*	Banuett and Herskowitz (1989)
SG200	*a1 mfa2 bE1bW2*	Kämper *et al*. (2006)
FB1 [pCM768]	*a1 b1 [pCM768]*	This work
FB2 [pCM768]	*a2 b2 [pCM768]*	This work
FB2 [pCMas-um02114]	*a2 b2 [pCMas-um02114]*	This work
FB1 [pCMas-um02125]	*a1 b1 [pCMas-um02125]*	This work
FB1 [pCMas-um02150]	*a1 b1 [pCMas-um02150]*	This work
FB1 [pCMas-um02151]	*a1 b1 [pCMas-um02151]*	This work
FB2 [pCMas-um10002]	*a2 b2 [pCMas-um10002]*	This work
FB2 [pCMas-um12232]	*a2 b2 [pCMas-um12232]*	This work
SG200Δ*um02151*	*a1 mfa2 bE1bW2 Δum02151*	This work
SG200ΔP*_as-um02151_*	*a1 mfa2 bE1bW2 Δ*P*_as-um02151_*	This work
SG200Δ*ncRNA1*	*a1 mfa2 bE1bW2 ΔncRNA1*	Morrison *et al*. (2012)
***Ustilago hordei***		
Uh 4857-4 (alias Uh364)^a^	*MAT-1*	Linning *et al*. (2004)
Uh 4857-5 (alias Uh365)	*MAT-2*	Linning *et al*. (2004)

**a.** Reference strain used for genome sequencing.

*Ustilago maydis* deletion strains were created by replacing the locus of interest with a Hyg^R^ cassette following a PCR-based approach (Kämper, 2004). To delete *um02151*, 1.1 kb fragments flanking *um02151* were amplified by PCR using primers um02151_Left_F and um02151_Left_R_SfiI to amplify the left flank, and primers um02151_Right_F_SfiI and um02151_Right_R to amplify the right flank (Table S5). To delete putative regulatory elements for *as-um02151*, a 0.5 kb region upstream from *as-um02151* (P*_as-um02151_*) was targeted for deletion. Primers pA_Left_F and pA_Left_R_SfiI, and pA_Right_F_SfiI and pA_Right_R were used to amplify 1.1 kb flanking regions to P*_as-um02151_* (Table S5). PCR fragments were digested with SfiI and ligated to the Hyg^R^ cassette as described in Morrison *et al*. (2012). The 4.8 kb ligation products were amplified using primers um02151_Nested_F and um02151_Nested_R, or pA_Nested_F and pA_Nested_R (Table S5), and cloned into pCR-XL-TOPO (Invitrogen) yielding pCR-XL-TOPO-Δum02151, and pCR-XL-TOPO-ΔP_as-um02151_ (Table S6). These vectors, carrying the deletion constructs, were transformed into One Shot Chemically Competent TOP10 *Escherichia coli* cells (Invitrogen). Nucleotide sequences were verified by sequencing using primers pMF1_HygOUT_F and pMF1_HygOUT_R. The *um02151* or P*_as-um02151_* deletion constructs were amplified by PCR using primers um02151_Nested_F and um02151_Nested_R, or pA_Nested_F and pA_Nested_R, and then purified using the QIAquick Gel Purification kit (Qiagen) prior to *U. maydis* transformation. *U. maydis* SG200 protoplasts were transformed. To identify putative transformants, PCRs were performed to amplify a region containing part of the Hyg^R^ cassette and an area of the flanking *um02151* or P*_as-um02151_* locus, outside the area of integration. For these screens, PCR was conducted using primer pairs um02151_Left_F and pMF1_HygOUT_F, and pMF1_HygOUT_R and um02151_Right_R, or primer pairs pA_Left_F and pMF1_HygOUT_F, and pMF1_HygOUT_R and pA_Right_R (Table S5). Several strains carrying deletions to *um02151* or P*_as-um02151_* were identified by PCR.

### Fungal strains, growth conditions and production of *U. maydis* cell types

*Ustilago maydis* and *U. hordei* strains used in this study are listed in Table 4. For RT-PCRs conducted to support EST data, *U. maydis* haploid strains FB1 and FB2 were grown in complete (CM) or minus nitrogen (MN) medium as described in Ho *et al*. (2010), filamentous growth of FBD12 diploids or FB1×FB2 dikaryons was induced as described in Morrison *et al*., (2012), and teliospores were harvested from mature tumours of corn (*Z. mays* L. ‘Golden Bantam’) infected with 518×521. Cob infection, teliospore harvesting and teliospore storage were performed as described in Morrison *et al*., (2012). For all other experiments: *U. maydis* haploid cells were grown overnight in YEPS medium (1% w/v yeast extract, 2% w/v peptone, 2% w/v sucrose; 250 r.p.m., 28°C). *U. maydis* teliospores were induced to germinate by modifying the protocol described in Zahiri *et al*. (2005). Differences include: germination was induced by shaking in 250 ml Erlenmeyer flasks containing 10 ml of YEPS medium, supplemented with 160 μg ml^−1^ streptomycin sulphate (90 r.p.m., 28°C). The germinating teliospores were pelleted 16, 18, 24 or 40 h post induction of germination (PIG) by centrifugation (400 *g*, 5 min, RT).

*Ustilago hordei* haploid strains Uh364 and Uh365, and barley (*Hordeum vulgare* L. ‘Odessa’) heads infected with Uh364×Uh365 were obtained from Guus Bakkeren (Agriculture & Agri-Food Canada, Pacific Agri-Food Research Centre, BC, Canada). Haploid cells were grown for ∼ 40 h in YEPS medium (250 r.p.m., 22°C). Mixtures of *U. hordei* teliospores, collected from field samples across Manitoba and eastern Saskatchewan in 2007 or 2009, were provided by James Menzies (Agriculture & Agri-Food Canada, Cereal Research Centre, MB, Canada). Plant growth conditions and pathogenesis assays were performed following the protocols described in Morrison *et al*. (2012).

### RNA extraction, S1 nuclease trimming and reverse transcription

RNA extractions for RT-PCRs conducted to support EST data were carried out on pelleted haploid cells grown in CM or MN, or filamentous diploids or dikaryons harvested from plates. The tissue from haploid or mycelial cell types was frozen in liquid nitrogen and homogenized using a mortar and pestle. Vacuum-dried teliospores were disrupted following the protocol described in Zahiri *et al*. (2005). For all other experiments, *U. maydis* haploid cells, *U. maydis* germinating teliospores, or *U. hordei* haploid cells, were pelleted by centrifugation. These cells, or *U. hordei* teliospores (collected from field samples), were resuspended in TRIzol reagent (Invitrogen), and transferred into 2 ml screw-cap tubes containing Lysing Matrix C (MP Biomedicals). Cells were disrupted as described in Zahiri *et al*. (2005). Alternatively, *U. hordei-*infected barley heads were ground in liquid nitrogen immediately prior to RNA extraction and resuspended in TRIzol reagent (Invitrogen). Following cell disruption, RNA was isolated using TRIzol reagent (Invitrogen) according to the manufacturer's protocol. RNA samples were precipitated, treated with DNase I, screened for genomic DNA contamination and assessed for quality as described in Morrison *et al*. (2012).

For first-strand synthesis reactions, 200 ng of DNase I-treated total RNA was used as template. These reactions were primed with oligo-d(T)_16_, a sense-specific primer, an antisense-specific primer, a tagged strand-specific primer or water (to account for false-priming). Primers were designed for sense- or antisense-specific first-strand synthesis reactions using Primer3 (Rozen and Skaletsky, 2000) following the protocol outlined in Ho *et al*. (2010). The strand-specific primer sequences are listed in Table S5. All first-strand synthesis reactions were carried out using the TaqMan Gold RT-PCR kit (Applied Biosystems) with the conditions outlined in Ho *et al*. (2010). Following first-strand synthesis, cDNA was diluted fourfold (1:3) with DEPC-treated water.

To detect dsRNA, 2.5 μg of DNase I-treated total RNA was incubated in S1 nuclease digestion reactions at 37°C for 30 min. In separate reactions, the final concentration of S1 Nuclease (Invitrogen) included: 0, 0.01, 0.1 or 1 U μl^−1^. Next, dsRNA was phenol/chloroform extracted, precipitated with NH_4_Ac/Ethanol/GlycoBlue Coprecipitant (Invitrogen) at −20°C for 60 min, and resuspended in 15 μl DEPC-treated H_2_O. Two microlitres of S1 trimmed RNA was used as template in first-strand synthesis reactions.

### Transcript expression analysis

All primers used in RT-PCRs to analyse transcript expression were designed using Primer3 (Rozen and Skaletsky, 2000) and are listed in Table S5. Two microlitres of diluted cDNA was used as template for each RT-PCR. All RT-PCRs were performed using Amplitaq Gold DNA polymerase (Applied Biosystems) and 30 or 35 cycles. RT-PCRs with 30 cycles were considered to be semi-quantitative because equal amounts of total RNA were used as template for each first-strand synthesis reaction, and equal volumes of cDNA were used as template for each RT-PCR. One quarter of the final product mixture was electrophoretically separated on an agarose gel (1× TAE), and visualized by ethidium bromide staining.

TaqMan minor groove binder (MGB) probes were designed using Primer Express v2.0 (Applied Biosystems) and are listed in Table S5. Two microlitres of diluted cDNA was used as template for each quantitative PCR (RT-qPCR). All RT-qPCRs were performed using the TaqMan Universal PCR Master Mix (Applied Biosystems). Data were collected and analysed on an ABI PRISM 7900HT using Sequence Detection System Version 2.1 (Applied Biosystems). Relative transcript levels for *um02151* were measured for eight independent FB1 [pCMas-um02151] strains, compared with an average of four independent FB1 [pCM768] strains using the ΔΔCT method. Three technical replicates for each sample were performed and *umgapd* was used as an internal standard.

### RNA ligase-mediated rapid amplification of cDNA ends (RLM-RACE)

RLM-RACE was employed to identify the 5’ and 3’ ends of *as-um02150*, *as-um02151* and *as-uhor_03676*. Ten micrograms of DNase I-treated total RNA from *U. maydis* (518×521) or *U. hordei* (Uh364×Uh365) teliospores was processed and reverse transcribed using the FirstChoice RLM-RACE kit (Ambion). The resulting cDNAs, containing 5’ or 3’ adaptors, were used as template for RACE. Successive PCRs utilizing gene-specific outer primers, followed by gene-specific inner primers (Table S5), were performed to yield 5’ or 3’ RACE products. All PCRs were conducted using HotStar HiFidelity DNA Polymerase (Qiagen). Amplified fragments were cloned into the pDrive Cloning Vector (Qiagen) and transformed into *E. coli* (Qiagen EZ Competent Cells). Transformants were plated on LB agar plates containing 100 μg ml^−1^ ampicillin, 50 μM isopropyl β-d-thiogalactopyranoside (IPTG) and 80 μg ml^−1^ 5-bromo-4-chloro-3-indolyl β-d-galactopyranoside (X-gal), which enabled blue/white screening of recombinant colonies. Following overnight growth at 37°C, 24 individual *E. coli* colonies from each transformation were inoculated into 96-well microtitre plates containing LB medium supplemented with 100 μg ml^−1^ ampicillin, and grown overnight at 250 r.p.m., 37°C. Portions of each bacterial culture were aliquoted into a separate 96-well microtitre plate and stored frozen at −80°C in 15% glycerol. The remaining bacterial cells were harvested by centrifugation and plasmid DNA was isolated using the protocol outlined in Nugent *et al*., (2004). Nucleotide sequences were determined using the primer M13_ F (Table S5).

### Protein extraction and analysis

Proteins were extracted from haploid cells grown in 15 ml YEPS medium, or YEPS medium supplemented with 250 μg ml^−1^ hygromycin B (Bioshop Canada). Cells were pelleted by centrifugation (5250 *g*, 5 min, 4°C), and washed once in 1 ml ice-cold protein extraction buffer [10 mM KCl, 5 mM MgCl_2_·6H_2_O, 400 mM sucrose, 100 mM Tris-HCl pH 8.1, 10% v/v glycerine, 0.007% v/v β-mercaptoethanol, 1% v/v Protease Inhibitor Cocktail (Sigma-Aldrich)]. Cells were resuspended in 300 μl ice-cold protein extraction buffer, transferred to 2 ml screw-cap tubes containing acid-washed glass beads (425–600 μm, Sigma-Aldrich), and disrupted as described in Zahiri *et al*. (2005). Cell debris was pelleted by centrifugation (20 800 *g*, 5 min, 4°C), the supernatant was transferred to a 1.5 ml microcentrifuge tube, and the total protein extract was stored at −20°C.

Protein concentrations were estimated using the Quick Start Bradford Protein Assay (Bio-Rad). The standard microplate protocol was followed. A standard curve was created using a bovine γ-globulin dilution series. For each sample, the absorbance at 595 nm was measured in triplicate, using a NanoDrop 8000 Spectrophotometer (Thermo Scientific). Total protein extracts were diluted to ∼ 1000 μg ml^−1^ and these concentrations were confirmed using the Bradford assay.

To visualize the total protein extract, SDS-PAGE was performed, followed by Coomassie staining. Briefly, equal parts of the diluted total protein extract was combined with Laemmli Sample Buffer (supplemented with 5% v/v β-mercaptoethanol), and this mixture was boiled for 5 min. Approximately 10 μg total protein extract was loaded into the wells of a 12% Mini-PROTEAN TGX precast polyacrylamide gel (Bio-Rad). Gels were stained with Coomassie Staining Solution (Sambrook and Russell, 2001), confirming equivalent amounts of total protein were present in the ∼ 1000 μg ml^−1^ dilutions.

For detection of Um02151, a Western blot was performed. Following SDS-PAGE, proteins were transferred electrophoretically onto a 0.2 μm PVDF membrane (Bio-Rad). Subsequently, the membrane was stained with Ponceau S (Sigma-Aldrich) to verify equal protein loading in each well. Prior to blocking, the Ponceau S stain was washed off the membrane using distilled water. The primary antibody (1:1000 dilution) used to probe the membrane was a rabbit affinity-purified polyclonal antibody against the Um02151 peptide APGKTKEDTLESLRC (GenScript). Binding of the primary antibody to the membrane was detected using a secondary antibody (1:3000 dilution) consisting of goat anti-rabbit immunoglobulin G, conjugated to alkaline phosphatase (Bio-Rad), in conjunction with the Immun-Blot Assay kit (Bio-Rad). An image of the Western blot was taken using the Geliance 600 Imaging System (Perkin Elmer). To estimate Um02151 levels, the pixel volume for each sample was determined using GeneTools software (Perkin Elmer). An average pixel volume was calculated for three biological replicates of the empty vector transformant, FB1[pCM768]. Using this average as a reference, the relative pixel volume for each sample was determined. The Western blot was repeated and the relative pixel volumes were separately calculated. The ‘fold change, relative to controls’ reported for each sample in Fig. 7B is an average of the values obtained from the two technical replicates.
