# Supplementary material for: Ustilago maydis natural antisense transcript expression alters mRNA stability and pathogenesis
Source: Mol Microbiol. 2013 May 30;89(1):29–51. doi: 10.1111/mmi.12254 (PMC3739942; doi:10.1111/mmi.12254)
Supplement: Supplementary file 1 [file mmi0089-0029-SD1.pdf]

Online Supporting Information

Ustilago maydis natural antisense transcript expression alters mRNA stability and pathogenesis

Michael E. Donaldson[1], and Barry J. Saville[1,2*]

[1]*Environmental and Life Sciences Graduate Program and*
[2]*Forensic Science Program, Trent University, Peterborough, ON, Canada K9J 7B8*
[*]For correspondence. E-mail barrysaville@trentu.ca, Tel. (+1) 705 748 1011 X7260

This PDF file includes:

Table S1-S6
Fig. S1-S2

Table S1. Putative NATs annotated through full-length cDNA sequence analysis.

NAT	cDNA library source[a]	NAT length (nt)	Sense transcript accession[b]	Type of NAT/ORF overlap	Length of NAT/ORF overlap (nt)	ORF contains intron	NAT feature	Putative ORFs >50aa <100aa	Putative ORFs >100aa
as01-um00047.2	DIK	1501	*um00047.2*	5' end	121	No	3' end of NAT also overlaps um00048 on sense strand	3	6
as01-um00067	DIK	1626	*um00067*	Embedded	1626	No		6	1
as01-um00133	D12	642	*um00133*	Embedded	522	Yes	NAT overlaps entire sense mRNA intron	1	0
as02-um00133	HCM	655	*um00133*	Embedded	535	Yes	NAT overlaps entire sense mRNA intron	1	0
as03-um00133	HCM	659	*um00133*	Embedded	539	Yes	NAT overlaps entire sense mRNA intron	1	0
as04-um00133	HCM	655	*um00133*	Embedded	535	Yes	NAT overlaps entire sense mRNA intron	1	0
as05-um00133	HCM	658	*um00133*	Embedded	538	Yes	NAT overlaps entire sense mRNA intron	1	0
as06-um00133	HCM	658	*um00133*	Embedded	538	Yes	NAT overlaps entire sense mRNA intron	1	0
as07-um00133	HCM	666	*um00133*	Embedded	546	Yes	NAT overlaps entire sense mRNA intron	1	0
as08-um00133	MMC	594	*um00133*	Embedded	474	Yes	NAT overlaps entire sense mRNA intron	1	0
as09-um00133	MMN	650	*um00133*	Embedded	530	Yes	NAT overlaps entire sense mRNA intron	1	0
as01-um00169	DIK	1165	*um00169*	Embedded	1154	Yes	NAT overlaps one sense mRNA intron splice junction	3	2
as01-um00329	HCM	862	*um00329*	Embedded	862	No		1	0
as01-um00442	HCM	441	*um00442*	3' end	246	No		0	0
as01-um00501	TDO	460	*um00501*	5' end	100	No		1	0
as01-um00513	TDO	210	*um00513*	Embedded	210	No		0	0
as01-um00712	TDO	503	*um00712*	Embedded	503	No		1	0
as01-um00743	MMN	496	*um00743*	Embedded	496	No		0	0
as01-um00751	MMN	929	*um00751*	Embedded	929	No		3	1
as01-um00784.2	D12	1440	*um00784.2*	5' end	1335	No		2	0
as01-um00842	HCM	927	*um00842*	3' end	780	No		1	3
as01-um00872	D12	1341	*um00872*	5' end	1057	No		8	0
as01-um00913	MMN	412	*um00913*	3' end	395	No		1	0
as02-um00913	TDO	659	*um00913*	3' end	397	No		2	0
as03-um00913	TDO	623	*um00913*	3' end	394	No		1	0
as01-um00947	DIK	1653	*um00947*	Embedded	1653	No		4	2
as01-um01110	DIK	1608	*um01110*	Embedded	1608	No		7	4
as01-um01216.2	MMN	959	*um01216.2*	Embedded	959	No		5	3
as01-um01252	DIK	1016	*um01252*	Embedded	1016	No		1	1
as01-um01374	T11	1100	***um01374***[c]	5' end	496	No		2	1
as01-um01386	HCM	161	*um01386*	3' end	79	Yes		0	0
as01-um01414	MMN	308	*um01414*	5' end	75	No		0	0
as01-um01439	T11	1620	*um01439*	3' end	1346	Yes	NAT overlaps one sense mRNA intron and an additional sense mRNA intron splice junction	3	1
as01-um01475	TDO	669	*um01475*	Embedded	669	Yes		1	0
as01-um01495	D12	1591	*um01495*	5' end	618	No		8	3
as01-um01624	HCM	160	*um01624*	5' end	96	Yes		0	0
as01-um01627	DIK	858	*um01627*	5' end	367	Yes	NAT overlaps one sense mRNA intron	3	1

							splice junction		
as01-um01670	TDO	2463	*um01670*	3' end	2372	No		6	10
as01-um01796	HCM	770	***um01796***[c]	3' end	374	No	NAT contains one intron (347 nt)	1	0
as01-um01832	MMN	234	*um01832*	Embedded	234	No		0	0
as01-um01977	MMC	880	*um01977*	3' end	841	No		1	1
as01-um02075	HCM	294	*um02075*	Embedded	294	No		0	0
as01-um02097	HCM	616	*um02097*	3' end	408	Yes	NAT overlaps one sense mRNA intron and an additional sense mRNA intron splice junction	0	0
as01-um02114	TDO	1166	*um02114*	Entire	1011	No		0	1
as01-um02123	DIK	1075	*um02123*	Embedded	1075	No		3	1
as02-um02123	TDO	551	*um02123*	Embedded	551	No		0	0
as01-um02125	TDO	1437	*um02125*	5' end	1429	No		2	1
as01-um02150	TDO	649	*um02150*	Embedded	649	No		0	2
as01-um02151	TDO	458	*um02151*	3' end	439	No		1	0
as01-um02168.2	MMN	1024	*um02168.2*	3' end	904	No	NAT contains one intron (97 nt)	7	1
as01-um02169	D12	1544	*um02169*	Entire	1191	No		7	0
as01-um02460	D12	1296	*um02460*	Embedded	1296	No		5	0
as01-um02496	TDO	997	*um02496*	Embedded	997	No		2	0
as01-um02523	DIK	1557	*um02523*	5' end	338	Yes	NAT overlaps entire sense mRNA intron	2	0
as01-um02594.2	MMN	526	*um02594.2*	Embedded	526	No		0	0
as01-um02640	TDO	204	*um02640*	Embedded	142	Yes	NAT overlaps one sense mRNA intron splice junction	0	0
as01-um02645	HCM	489	*um02645*	3' end	28	Yes	NAT overlaps one sense mRNA intron splice junction	2	1
as01-um02729	MMN	959	*um02729*	Embedded	959	No		7	2
as01-um02742	MMN	346	*um02742*	5' end	35	Yes	NAT overlaps one sense mRNA intron splice junction and NAT contains one intron (1000 nt)	1	0
as01-um02789	HCM	630	*um02789*	3' end	253	Yes	NAT overlaps two sense mRNA introns entirely	3	0
as02-um02794	DIK	1112	*um02794*	3' end	608	No		2	0
as01-um02794	MMN	523	*um02794*	5' end	141	No		0	0
as03-um02794	T11	905	*um02794*	5' end	560	No		2	0
as04-um02794	T11	615	*um02794*	5' end	560	No		0	0
as01-um02803	TDO	620	*um02803*	Embedded	620	No		1	0
as01-um02827	D12	1182	*um02827*	Embedded	1182	No		2	2
as01-um02828	D12	926	*um02828*	5' end	910	No		3	1
as01-um02851	DIK	894	*um02851*	Entire	606	No		1	0
as01-um02925	MMN	352	*um02925*	5' end	80	Yes	NAT overlaps one sense mRNA intron splice junction	0	0
as01-um02993.2	TDO	487	*um02993.2*	Embedded	487	No		1	0
as01-um03175	D12	1133	*um03175*	Embedded	1133	No		4	3
as01-um03232	MMN	532	*um03232*	5' end	228	No		1	1
as01-um03240	DIK	683	*um03240*	5' end	187	No		3	1
as01-um03263	DIK	1414	*um03263*	3' end	1412	No		3	2

as01-um03267	TDO	1357	*um03267*	5' end	1011	No		3	2
as01-um03283	MMC	463	*um03283*	Embedded	371	Yes	NAT overlaps entire sense mRNA intron	0	0
as01-um03351	TDO	714	*um03351*	5' end	459	No		2	1
as01-um03365	MMN	245	*um03365*	3' end	206	No		0	0
as01-um03372	TDO	594	*um03372*	Embedded	560	Yes	NAT overlaps one sense mRNA intron splice junction	0	0
as01-um03413	MMN	653	*um03413*	Embedded	653	No		0	0
as01-um03458	TDO	999	*um03458*	3' end	752	No		0	0
as01-um03476	DIK	469	*um03476*	3' end	290	No		4	0
as01-um03507	D12	1062	*um03507*	5' end	792	No		3	0
as01-um03613	D12	1774	***um03613***[c]	Embedded	1774	No		6	1
as01-um03618	MMN	745	*um03618*	3' end	694	No		4	2
as01-um03621	TDO	261	*um03621*	Embedded	261	No		0	0
as01-um03630	DIK	1233	*um03630*	Entire	870	No		0	1
as01-um03645	TDO	1084	*um03645*	Embedded	1084	No		1	0
as01-um03664	MMN	453	*um03664*	5' end	416	No		0	0
as01-um03751	HCM	443	***um03751***[c]	3' end	389	No		0	0
as02-um03798	MMN	286	*um03798*	3' end	286	No		0	0
as01-um03798	T11	890	*um03798*	Embedded	667	No		0	0
as01-um03841	MMN	536	*um03841*	3' end	294	No		0	0
as01-um03872	MMN	409	*um03872*	3' end	39	No		0	0
as01-um03898	TDO	1832	*um03898*	3' end	1387	No		2	2
as01-um03914	HCM	1192	*um03914*	3' end	1121	No		0	2
as01-um03917.2	MMN	446	*um03917.2*	3' end	155	No		0	0
as01-um03947	TDO	326	*um03947*	3' end	199	No		0	0
as01-um03948	MMN	637	*um03948*	Embedded	505	Yes	NAT overlaps one sense mRNA intron and an additional sense mRNA intron splice junction	1	0
as01-um04059	MMN	331	*um04059*	Embedded	331	No		1	0
as01-um04061	MMN	1624	*um04061*	5' end	886	Yes	NAT overlaps one sense mRNA intron and an additional sense mRNA intron splice junction	4	4
as01-um04236	DIK	469	*um04236*	3' end	439	No		2	0
as01-um04259	MMN	649	*um04259*	Embedded	649	No		1	0
as01-um04278.2	DIK	861	*um04278.2*	5' end	215	No		1	0
as02-um04278.2	DIK	861	*um04278.2*	5' end	215	No		1	0
as01-um04327	TDO	314	*um04327*	Embedded	314	No		0	0
as01-um04329.2	MMN	496	*um04329.2*	Embedded	496	No		2	0
as01-um04342	TDO	833	*um04342*	5' end	518	No		3	0
as01-um04354	TDO	618	*um04354*	3' end	483	No		1	1
as01-um04361	MMC	649	*um04361*	Embedded	649	No		2	0
as01-um04385.2	DIK	1582	*um04385.2*	5' end	569	No		3	3
as01-um04397	DIK	1404	*um04397*	Embedded	1404	Yes		4	2
as01-um04426	T11	690	*um04426*	3' end	456	No		1	0
as01-um04448	T11	484	*um04448*	3' end	104	No		1	0
as01-um04466	DIK	1157	*um04466*	5' end	859	No		2	1

as01-um04495	TDO	410	*um04495*	Embedded	410	Yes		0	0
as01-um04654	MMN	582	*um04654*	3' end	340	No		1	0
as01-um04711	MMN	1106	*um04711*	3' end	1016	No		1	4
as01-um04844.2	MMN	234	*um04844.2*	Embedded	234	No		0	0
as01-um04852	MMN	1017	*um04852*	Embedded	1017	No		1	0
as01-um04878	D12	1715	*um04878*	Embedded	1715	No		3	3
as01-um04936	TDO	663	***um04936***[c]	3' end	601	No		0	0
as02-um04936	TDO	477	***um04936***[c]	Embedded	477	No		0	0
as01-um04961	HCM	746	*um04961*	Embedded	746	No		1	1
as01-um05019	TDO	608	*um05019*	5' end	112	Yes	NAT overlaps one sense mRNA intron splice junction	0	1
as01-um05082	MMN	725	*um05082*	5' end	577	No		1	0
as01-um05107	TDO	362	*um05107*	Embedded	362	No		0	0
as01-um05118	DIK	1738	*um05118*	3' end	1247	Yes	NAT overlaps entire sense mRNA intron	5	2
as01-um05131	TDO	757	*um05131*	Embedded	757	Yes		1	0
as01-um05243	TDO	206	*um05243*	Embedded	53	Yes	NAT overlaps two splice junctions for separate sense mRNA introns (overlaps an entire exon)	0	0
as01-um05271.2	DIK	1164	*um05271.2*	5' end	774	No		7	2
as01-um05326	TDO	643	*um05326*	Embedded	643	No		1	0
as01-um05363	HCM	582	*um05363*	5' end	546	No		1	0
as01-um05377	TDO	662	*um05377*	3' end	658	No		1	0
as01-um05396.2	HCM	629	*um05396.2*	3' end	588	No		2	0
as01-um05399	MMN	646	*um05399*	3' end	282	No		0	0
as02-um05399	MMN	327	*um05399*	3' end	282	No		0	0
as03-um05399	MMN	245	*um05399*	Embedded	245	No		0	0
as01-um05401	TDO	585	*um05401*	Embedded	585	No		0	2
as01-um05454	DIK	779	*um05454*	Embedded	779	No		3	1
as01-um05475	MMN	738	*um05475*	5' end	330	No		1	0
as01-um05486	TDO	906	*um05486*	Embedded	906	No		1	5
as02-um05486	TDO	523	*um05486*	Embedded	523	No		0	0
as01-um05589	TDO	483	*um05589*	5' end	446	No		3	0
as02-um05589	TDO	543	*um05589*	Entire	450	No		5	1
as01-um05648	TDO	923	*um05648*	Embedded	923	No		2	0
as01-um05702	TDO	517	*um05702*	Embedded	517	No		1	0
as01-um05725	D12	919	*um05725*	5' end	675	No		0	0
as01-um05731	HCM	752	***um05731***[c]	5' end	313	No		3	0
as01-um05737	D12	845	*um05737*	5' end	551	No		3	0
as01-um05880	MMN	458	*um05880*	3' end	381	No		1	0
as01-um05936	MMN	469	*um05936*	3' end	418	No		0	1
as01-um05989	HCM	646	*um05989*	Embedded	646	No		0	1
as01-um06045	TDO	750	*um06045*	3' end	734	No		0	0
as01-um06130	DIK	1560	*um06130*	Embedded	1560	No		6	0
as01-um06164	TDO	1069	*um06164*	Embedded	1069	No		2	3
as01-um06188	HCM	880	*um06188*	Embedded	880	No		0	0
as01-um06273	TDO	1043	*um06273*	5' end	139	No		3	0

as01-um06390	D12	2867	*um06390*	Entire	450	No	3' end of NAT also overlaps um06391 on sense strand	3	13
as01-um06431	TDO	669	*um06431*	3' end	608	Yes		1	1
as01-um10000	DIK	1699	*um10000*	Embedded	1699	No		2	0
as01-um10002	DIK	1116	*um10002*	3' end	1101	No		3	4
as01-um10013.2	T11	391	*um10013.2*	3' end	42	No		0	0
as01-um10018	DIK	1152	*um10018*	3' end	1035	No		4	0
as01-um10027	T11	601	*um10027*	Embedded	601	Yes		2	0
as02-um10027	TDO	562	*um10027*	Embedded	562	Yes		2	0
as03-um10027	TDO	444	*um10027*	Embedded	444	Yes		2	0
as04-um10027	TDO	494	*um10027*	Embedded	494	Yes	NAT contains two introns (386 and 194 nt)	1	0
as05-um10027	TDO	337	*um10027*	Embedded	337	Yes		0	0
as06-um10027	TDO	575	*um10027*	Embedded	575	Yes		2	0
as07-um10027	TDO	572	*um10027*	Embedded	572	Yes		2	0
as01-um10062	T11	911	*um10062*	Embedded	879	Yes	NAT overlaps one sense mRNA intron splice junction	3	0
as01-um10078	DIK	1160	*um10078*	5' end	362	No	3' end of NAT also overlaps um10079 on antisense strand	2	1
as01-um10148	T11	787	*um10148*	3' end	380	Yes		4	0
as02-um10148	MMC	984	*um10148*	3' end	538	Yes		4	0
as01-um10155	MMN	853	*um10155*	5' end	735	No		3	1
as01-um10193	TDO	345	*um10193*	3' end	92	No		0	0
as01-um10220	MMN	919	*um10220*	Embedded	919	Yes		2	0
as02-um10220	MMN	919	*um10220*	Embedded	919	Yes		2	0
as01-um10306	MMN	317	*um10306*	3' end	151	No		0	0
as01-um10308	TDO	182	*um10308*	5' end	27	No		0	0
as01-um10338	HCM	897	*um10338*	Embedded	897	No		1	2
as01-um10406	DIK	672	*um10406*	3' end	488	No		1	0
as01-um10473	HCM	610	*um10473*	5' end	252	No		1	0
as01-um10533	TDO	480	*um10533*	5' end	35	No		0	0
as01-um10538	MMN	507	*um10538*	Embedded	507	No		4	3
as01-um10606	D12	1564	*um10606*	Embedded	1564	No		3	1
as01-um10671	D12	295	*um10671*	Embedded	295	No		0	0
as01-um10751	MMN	661	*um10751*	Embedded	661	No		4	0
as01-um10792	MMN	542	*um10792*	5' end	297	No		0	0
as01-um10800	D12	1234	*um10800*	3' end	876	No		4	0
as01-um10808	MMN	232	*um10808*	Embedded	37	Yes	NAT overlaps two splice junctions for separate sense mRNA introns (overlaps an entire exon)	0	0
as01-um10817	MMN	253	*um10817*	Embedded	253	No		0	0
as01-um10881	MMC	834	*um10881*	Entire	387	Yes	NAT overlaps entire sense mRNA intron	0	0
as01-um10903	MMN	242	*um10903*	3' end	35	Yes	NAT overlaps one sense mRNA intron splice junction	0	0
as01-um10946	MMN	874	*um10946*	3' end	862	No		2	1
as01-um10964	DIK	889	*um10964*	5' end	637	No		3	2
as01-um11010	DIK	1785	*um11010*	5' end	1082	No	3' end of NAT also overlaps um11009 on	2	0

							antisense strand		
as01-um11125	MMN	258	*um11125*	5' end	92	No		0	0
as01-um11303	MMN	324	*um11303*	Embedded	324	No		0	0
as01-um11310	TDO	369	*um11310*	Embedded	369	No		0	0
as01-um11317	MMN	202	*um11317*	3' end	135	No		0	0
as01-um11383	DIK	1196	*um11383*	Embedded	1196	No		3	1
as01-um11560	TDO	653	*um11560*	Embedded	653	No		2	1
as01-um11585	MMN	876	*um11585*	3' end	745	No	NAT contains one intron (108 nt)	3	1
as01-um11603	T11	1108	*um11603*	Entire	522	Yes	NAT overlaps two sense mRNA introns entirely	2	1
as01-um11644	HCM	647	*um11644*	3' end	628	No		0	0
as01-um11668	DIK	2029	*um11668*	Embedded	2029	No		3	0
as01-um11689	TDO	530	*um11689*	5' end	82	Yes	NAT overlaps entire sense mRNA intron	1	0
as01-um11924	MMN	149	*um11924*	3' end	64	No		0	0
as01-um11941	DIK	892	*um11941*	3' end	349	No	NAT contains one intron(324 nt)	3	0
as01-um12007	HCM	226	*um12007*	Embedded	226	No		0	0
as01-um12043	TDO	612	*um12043*	Entire	411	No		1	0
as01-um12087	MMC	705	*um12087*	3' end	520	No		3	4
as01-um12195	MMN	231	*um12195*	Embedded	164	Yes	NAT overlaps one sense mRNA intron splice junction	0	0
as01-um12219	MMN	223	*um12219*	Embedded	223	No		0	0
as01-um12232	DIK	2057	*um12232*	3' end	2037	No		6	1
as01-um12291	TDO	1702	***um12291***[c]	Embedded	1702	Yes		3	5
as01-um15059	TDO	621	*um15059*	Embedded	621	Yes		0	2

[a]ESTs were represented in *U. maydis* cDNA libraries created from germinating (T11) and dormant (TDO) teliospores, haploid cells grown in complete medium (HCM), carbon starvation medium (MMC) or nitrogen starvation medium (MMN), forced diploids grown filamentously (D12), or filamentous dikaryotic mycelia (DIK).

[b]Accession according to MUMDB (http://mips.helmholtz-muenchen.de/genre/proj/ustilago/).

[c]Putative *U. maydis* effector proteins in bold font.

NAT: natural antisense transcript, nt: nucleotides, ORF: open reading frame, aa: amino acids.

Table S2. Features of putative peptides translated from ORFs within full-length NATs.

NAT	ORF start (nt)	ORF stop (nt)	Peptide length (aa)	Signal peptide length (aa)	Mature peptide length (aa)	Cysteins in mature peptide	SignalP score	TargetP RC	IP score	Best blastp alignment	blastp E-value
as01-um03175	572	946	124	32	92	2	0.597	4	9.6		
as01-um04259	23	295	90	16	74	2	0.454	3	6.6		
as01-um05271.2	922	1158	78	17	61	4	0.715	1	9.5		
as01-um06130	57	305	82	27	55	1	0.614	4	3.2		
as01-um10538	164	487	107	20	87	6	0.540	3	9.0		
as01-um10751	65	217	50	20	30	3	0.531	4	7.2		
as01-um11560	244	618	124	22	102	5	0.551	4	4.7		
as01-um12291	1226	1474	82	29	53	3	0.781	1	9.7		
as01-um00047.2	698	1447	249							*Ustilago maydis* hypothetical protein UM00048	1.0E-178
as01-um01110	272	634	120							*Candida albicans* hypothetical protein CaO19.1284	4.0E-17
as01-um03351	233	382	49							*Candida tenuis* hypothetical protein CANTEDRAFT_110083	1.0E-13
as01-um06390	815	2764	649							*Ustilago maydis* related to multidrug resistant protein UM06391	0.0E+00
as01-um10078	101	541	146							*Ustilago hordei* uncharacterized protein UHOR_03688	5.0E-13

NAT: natural antisense transcript, ORF: open reading frame, nt: nucleotides; aa: amino acids; IP: integral prediction; RC: reliability class.

Table S3. Putative NATs annotated through cDNA sequence analysis.

NAT	3'[a]	cDNA library source[b]	NAT length (nt)	Sense transcript accession[c]	Type of NAT/ORF overlap	Length of NAT/ORF overlap (nt)	ORF contains intron	NAT feature
as10-um00133	Yes	MMC	613	*um00133*	Embedded	493	Yes	NAT overlaps entire sense mRNA intron
as02-um00501	Yes	D12	891	*um00501*	5' end	764	No	
as01-um00755	Yes	D12	132	*um00755*	Embedded	132	No	
as01-um00933	Yes	D12	2634	*um00933*	Embedded	2634	No	
as01-um01047	Yes	MMN	561	*um01047*	5' end	63	No	
as01-um01668	Yes	TDO	563	*um01668*	3' end	527	No	
as01-um02375	Yes	DIK	795	*um02375*	3' end	511	No	
as01-um02403.2	Yes	HCM	397	*um02403.2*	Embedded	397	No	
as01-um02682	Yes	D12	1177	*um02682*	Embedded	1177	No	
as02-um02742	Yes	MMN	186	*um02742*	5' end	173	Yes	
as01-um04381	Yes	D12	2439	*um04381*	3' end	2421	No	
as01-um04959	Yes	DIK	1673	*um04959*	3' end	1567	No	
as01-um05014	Yes	TDO	541	*um05014*	Embedded	541	No	
as02-um05082	Yes	D12	1185	*um05082*	3' end	997	No	
as01-um05126	Yes	DIK	627	*um05126*	3' end	571	No	
as01-um05564	Yes	D12	2983	*um05564*	3' end	2924	Yes	
as01-um06001	Yes	D12	310	*um06001*	5' end	137	No	
as01-um06046	Yes	T11	754	*um06046*	5' end	534	No	
as01-um06062.2	Yes	D12	1807	*um06062.2*	Embedded	1807	No	
as01-um06281	Yes	D12	1508	*um06281*	5' end	911	Yes	NAT overlaps entire sense mRNA intron
as08-um10027	Yes	TDO	546	*um10027*	Embedded	546	Yes	
as01-um10213	Yes	HCM	957	*um10213*	Embedded	957	Yes	NAT and sense mRNA have same intron (656 nt)
as01-um10422	Yes	DIK	964	*um10422*	Embedded	964	No	
as01-um10681	Yes	D12	486	*um10681*	3' end	280	Yes	
as01-um10775	Yes	D12	1050	*um10775*	3' end	944	No	
as01-um10951	Yes	D12	1229	*um10951*	Entire	789	No	
as01-um11590	Yes	D12	500	*um11590*	3' end	334	Yes	
as01-um11594	Yes	D12	1324	*um11594*	5' end	853	No	
as11-um00133	No	HCM	616	*um00133*	Embedded	496	Yes	NAT overlaps entire sense mRNA intron
as12-um00133	No	MMC	631	*um00133*	Embedded	511	Yes	NAT overlaps entire sense mRNA intron
as13-um00133	No	HCM	107	*um00133*	Embedded	107	Yes	
as14-um00133	No	HCM	469	*um00133*	Embedded	357	Yes	NAT overlaps one sense mRNA intron splice junction
as15-um00133	No	MMC	463	*um00133*	Embedded	357	Yes	NAT overlaps one sense mRNA intron splice junction
as01-um00277	No	DIK	572	*um00277*	5' end	74	No	
as01-um00366	No	D12	389	*um00366*	3' end	234	No	
as01-um00605	No	DIK	400	*um00605*	3' end	345	No	
as01-um00625	No	DIK	265	*um00625*	3' end	39	No	
as02-um00751	No	D12	557	*um00751*	Embedded	557	No	
as01-um00757	No	HCM	602	*um00757*	Embedded	602	No	
as02-um00933	No	D12	460	*um00933*	Embedded	460	No	
as01-um01053	No	MMC	276	*um01053*	3' end	131	No	NAT contains one intron (156 nt)
as01-um01130	No	D12	503	*um01130*	5' end	460	No	

as01-um02765	No	MMN	566	*um02765*	Embedded	566	No	
as01-um03038	No	D12	577	*um03038*	3' end	336	No	
as01-um03097	No	MMC	476	*um03097*	3' end	339	No	
as01-um03113	No	D12	53	*um03113*	Embedded	53	No	
as01-um03570	No	D12	216	*um03570*	3' end	148	Yes	
as01-um03665	No	MMN	554	*um03665*	3' end	81	No	
as01-um04227	No	D12	537	*um04227*	Embedded	537	No	
as01-um05626	No	HCM	558	*um05626*	3' end	512	No	
as01-um05733	No	MMN	574	*um05733*	5' end	338	Yes	NAT and sense mRNA have same intron (158 nt) and NAT overlaps one additional sense mRNA intron splice junction
as01-um06117.2	No	D12	496	*um06117.2*	Embedded	496	Yes	
as02-um06281	No	D12	706	*um06281*	Embedded	610	Yes	NAT overlaps entire sense mRNA intron
as01-um06285	No	D12	544	*um06285*	Embedded	544	No	
as02-um10155	No	D12	504	*um10155*	5' end	428	No	
as03-um10155	No	HCM	502	*um10155*	Embedded	502	No	
as01-um10210.2	No	HCM	521	*um10210.2*	3' end	193	Yes	
as01-um10273	No	MMN	560	*um10273*	5' end	136	Yes	
as01-um10712	No	HCM	218	*um10712*	3' end	195	Yes	NAT and sense mRNA have same intron (168 nt)
as01-um10870	No	DIK	684	*um10870*	3' end	96	No	
as01-um10882	No	HCM	218	*um10882*	3' end	168	Yes	NAT overlaps one sense mRNA intron splice junction and NAT contains one intron (164 nt)
as02-um10882	No	MMN	343	*um10882*	3' end	191	Yes	NAT overlaps entire sense mRNA intron
as01-um10939	No	DIK	502	*um10939*	Embedded	502	No	
as02-um10939	No	DIK	502	*um10939*	Embedded	502	No	
as01-um11048	No	DIK	432	*um11048*	3' end	157	No	
as01-um11165	No	D12	277	*um11165*	Embedded	277	No	
as01-um11382	No	D12	574	*um11382*	Embedded	169	Yes	NAT overlaps one sense mRNA intron splice junction
as01-um11472	No	D12	69	*um11472*	Embedded	69	No	
as01-um11517	No	HCM	496	*um11517*	5' end	480	Yes	NAT and sense mRNA have same intron (131 nt)
as01-um11563	No	D12	537	*um11563*	3' end	321	Yes	
as01-um11696	No	D12	937	*um11696*	3' end	770	Yes	
as02-um12043	No	HCM	400	*um12043*	3' end	375	No	
as01-um15066	No	D12	531	*um15066*	Embedded	531	Yes	

[a] 3' sequence available (but lacks a poly(A) tail).

[b] ESTs were represented in *U. maydis* cDNA libraries created from germinating (T11) and dormant (TDO) teliospores, haploid cells grown in complete medium (HCM), carbon starvation medium (MMC) or nitrogen starvation medium (MMN), forced diploids grown filamentously (D12), or filamentous dikaryotic mycelia (DIK).

[c] Accession according to MUMDB (http://mips.helmholtz-muenchen.de/genre/proj/ustilago/).

NAT: natural antisense transcript, nt: nucleotides, ORF: open reading frame.

Table S4. RT-PCR results confirming *in silico* analysis.

Locus	Remark
um00047	NAT detected in all cell-types, and nutritional conditions
um00133	NAT detected in all cell-types, and nutritional conditions
um00169	NAT detected in all cell-types, and nutritional conditions
um00751	NAT detected in all cell-types, and nutritional conditions
um00784.2	NAT detected in all cell-types, and nutritional conditions
um00842	NAT detected in all cell-types, and nutritional conditions
um01110	NAT detected in all cell-types, and nutritional conditions
um01252	NAT detected in all cell-types, and nutritional conditions
um01495	NAT detected in all cell-types, and nutritional conditions
um01796	NAT detected in all cell-types, and nutritional conditions
um02075	NAT detected in all cell-types, and nutritional conditions
um02168.2	NAT detected in all cell-types, and nutritional conditions
um02169	NAT detected in all cell-types, and nutritional conditions
um02794	NAT detected in all cell-types, and nutritional conditions
um03263	NAT detected in all cell-types, and nutritional conditions
um03413	NAT detected in all cell-types, and nutritional conditions
um03618	NAT detected in all cell-types, and nutritional conditions
um03630	NAT detected in all cell-types, and nutritional conditions
um03645	NAT detected in all cell-types, and nutritional conditions
um03798	NAT detected in all cell-types, and nutritional conditions
um03898	NAT detected in all cell-types, and nutritional conditions
um03914	NAT detected in all cell-types, and nutritional conditions
um04059	NAT detected in all cell-types, and nutritional conditions
um04061	NAT detected in all cell-types, and nutritional conditions
um04327	NAT detected in all cell-types, and nutritional conditions
um04654	NAT detected in all cell-types, and nutritional conditions
um05131	NAT detected in all cell-types, and nutritional conditions
um05271	NAT detected in all cell-types, and nutritional conditions
um05363	NAT detected in all cell-types, and nutritional conditions
um05454	NAT detected in all cell-types, and nutritional conditions
um05725	NAT detected in all cell-types, and nutritional conditions
um05989	NAT detected in all cell-types, and nutritional conditions
um06045	NAT detected in all cell-types, and nutritional conditions
um06130	NAT detected in all cell-types, and nutritional conditions
um06188	NAT detected in all cell-types, and nutritional conditions
um06285	NAT detected in all cell-types, and nutritional conditions
um10002	NAT detected in all cell-types, and nutritional conditions
um10027	NAT detected in all cell-types, and nutritional conditions
um10062	NAT detected in all cell-types, and nutritional conditions
um10671	NAT detected in all cell-types, and nutritional conditions
um10751	NAT detected in all cell-types, and nutritional conditions
um10792	NAT detected in all cell-types, and nutritional conditions
um10881	NAT detected in all cell-types, and nutritional conditions
um10946	NAT detected in all cell-types, and nutritional conditions
um11010	NAT detected in all cell-types, and nutritional conditions
um11590	NAT detected in all cell-types, and nutritional conditions
um11603	NAT detected in all cell-types, and nutritional conditions
um11644	NAT detected in all cell-types, and nutritional conditions
um11696	NAT detected in all cell-types, and nutritional conditions
um12087	NAT detected in all cell-types, and nutritional conditions
um10078	NAT detected in all cell-types, and nutritional conditions
um02125	NAT enriched in TDO
um02150	NAT enriched in TDO
um02151	NAT enriched in TDO

um04878	NAT enriched in TDO
um06164	NAT enriched in TDO
um12232	NAT enriched in TDO
um00913	NAT not detected in TDO
um04959	NAT not detected in TDO
um06117	NAT not detected in TDO
um00067	NAT enriched in HMN
um01668	NAT enriched in HMN
um05702	NAT not detected in HMN
um02097	NAT enriched in HCM, HMN, and DIK (not detected in diploid cell-types)
um05564	NAT enriched in HCM, HMN, and TDO (not detected in filamentous cells)
um01439	NAT not detected in any-cell type, or nutritional condition
um00329	High background (but NAT detected in at least one cell-type, or nutritional condition)
um00947	High background (but NAT detected in at least one cell-type, or nutritional condition)
um02403.2	High background (but NAT detected in at least one cell-type, or nutritional condition)
um04381	High background (but NAT detected in at least one cell-type, or nutritional condition)
um04397	High background (but NAT detected in at least one cell-type, or nutritional condition)
um04495	High background (but NAT detected in at least one cell-type, or nutritional condition)
um05126	High background (but NAT detected in at least one cell-type, or nutritional condition)
um05880	High background (but NAT detected in at least one cell-type, or nutritional condition)
um06281	High background (but NAT detected in at least one cell-type, or nutritional condition)
um10213	High background (but NAT detected in at least one cell-type, or nutritional condition)
um11941	High background (but NAT detected in at least one cell-type, or nutritional condition)
um00712	Inconclusive
um00872	Inconclusive
um01627	Inconclusive
um01670	Inconclusive
um02375	Inconclusive
um02523	Inconclusive
um02803	Inconclusive
um03038	Inconclusive
um03097	Inconclusive
um03351	Inconclusive
um03841	Inconclusive
um04361	Inconclusive
um04466	Inconclusive
um05014	Inconclusive
um05737	Inconclusive
um10018	Inconclusive
um11009	Inconclusive
um11585	Inconclusive
um11668	Inconclusive

[a]One NAT (*as-um01439*) was not detected in RT-PCR screens, but *as-um01439* overlaps a splice junction in *um01439* and *as-um01439* was identified during analysis of the germinating teliospore cDNA library (Ho *et al*., 2007). These observations suggested that *as-um01439* is annotated in the correct orientation and that it may be detected solely in the germinating teliospore, a cell-type not investigated in this RT-PCR screen.

Table S5. Primers used in this study.

Primer	Nucleotide sequence (5' to 3' direction)[a, b]
Ustilago maydis	
**Sequencing**	
M13_R	aaacagctatgaccatgttca
dT19V	tttttttttttttttttttv
M13_F	cccagtcacgacgttgtaaaacg
pgapd_79_F	GACCTCACTCTTCAAGAACAAGC
pCM768_Hyg_F	agtgcttgacattggggaac
pCM768_Hyg_R	gatgttggcgacctcgtatt
**Antisense expression vector creation**	
as-um02125_F_SphI	ggagcatgcGCGTCGCTGCTGATCTGCGT
as-um02125_R_HindIII	ggaaagcttCTTCAAAATGGCCGTTGGCGG
as-um02150_F_SphI	ggagcatgcACCACATCAACGACCACCACTCG
as-um02150_R_HindIII	ggaaagcttGCCAGGAAAAGTCGCTCCCAAC
as-um02151_F_BamHI	ggaggatccGTACACCAAAGGCGAACGATCACTC
as-um02151_R_HindIII	ggaaagcttGGAAGGCAAAGTCATCACCATTGG
as-um02114_F_BamHI	ggaggatccAGCCTCGAAATAGTTCAAATTCATAGCTGC
as-um02114_R_HindIII	ggaaagcttCACCGCGGAGAAAGCCC
as-um10002_F_HindIII	ggaaagcttGTCGCAGGTTTACGCTTAGAGCTTCT
as-um10002_R_HindIII	ggaaagcttACAAGACGTGGGGCGAGGG
as-um12232_F_BglII	ggaagatctGCCGAAGTCGAGCTGCTAACTTAG
as-um12232_R_BglII	ggaagatctGCTGAGCACAAGGAATTAACGACC
**SG200Δ*um02151* creation**	
um02151_Left_F	GCTACGAAGAAGAGTCCATAGTTGC
um02151_Left_R_SfiI	gttgggccatctaggccTCGTTCGCCTTTGGTGTACA
um02151_Right_F_SfiI	caacggcctgagtggccGCTTGCGGAATTTGAGAATG
um02151_Right_R	AATCATGCCAATATCGTCGTC
um02151_Nested_F	CGATCTAGCGTCTTTGGTTG
um02151_Nested_R	CTGTCTTCGTGCGTCTCAGC
pMF1_HygOUT_F	ctgcgcgtgtcgattcac
pMF1_HygOUT_R	tacgcctctaccgacaccac
**SG200$\Delta_{pas\text{-}um02151}$ creation**	
pA_Left_F	AGATCAACGTCACTGGCTCCTTCC
pA_Left_R_SfiI	gttgggccatctaggccGATTCACACTTCCCATGCCACG
pA_Right_F_SfiI	caacggcctgagtggccGCGACAATAACAAACCATCTCCCTG
pA_Right_R	TGCCTTACCTCCACTTGTGTCTTGG
pA_Nested_F	GCTGGCACCTACAAAACCTC
pA_Nested_R	TCGTCTCTCATCCTTCTTAGCC
**Strand-specific first strand synthesis**	
umgapd_FS	CCGAGATGACGACCTTCTTG
um01110_FS	ATCACCTCGATCGTGTCCTC
um02114_FS	AGCACCCTTTTCCTCGATTAC
um02125_FS	GCAAACATTCTCCTCGTCTTG
um02150_FS	GCACACCACTCTTTAGCGAAC
um02151_FS	CCTGATCCTTCGCATCTAGC
um02151_FS_NO	GTGTGGATGAGGAAGCAGTC
um04878_FS	AGCGAAACTCCTTCTCCTCTG
um06164_FS	TGCCACTCTGAGAATACTGCTG
um10002_FS	GACCTTTCTTCCACAGCCTTT
um12232_FS	TCATGTCAACTTCTGCTGCAC
as-um02114_FS	GATGGCAAGCTCATTGAGAAG

as-um00047_FS	TGCTAGAACGTCGCGTCTG
as-um00067_FS	CAGACTCGCTCCTTATTCGC
as-um00133_FS	TTGACATTCGAAAAGATTGGG
as-um00169_FS	AGACGAGTTTGTTGCGGC
as-um00329_FS	AAAGATCGCCGATAATGTGG
as-um00712_FS	GAGGTGGAGAACGATTCAGC
as-um00751_FS	TTTCGGTAGCTGAGCTTATGG
as-um00784.2_FS	CGAGTGACGATGACGATGAC
as-um00842_FS	TCGGTACTTGGGTCCTTCTG
as-um00872_FS	AAAGTACGGCGGTACCAGTG
as-um00913_FS	TTCGAACTGGATTTTCCACG
as-um00947_FS	CCGAGAATGGTCATTTCCAC
as-um01110_FS	TCGACGACTCAAACCATTCC
as-um01252_FS	GCTCTGCTTACATTCCCGAG
as-um01439_FS	TTACTATGGCCTTCTTCGCC
as-um01495_FS	GTCGTCGGTGGTCACTTGTC
as-um01627_FS	CGTTGGTGCTGTCCGATT
as-um01668_FS	AGCACACTTGCTGAATACGC
as-um01670_FS	TTGACATCGCAGATCCAGAC
as-um01796_FS	GTCGACATTCCTTCAGACCG
as-um02075_FS	CTTGAATGCGTGTGGGTCTC
as-um02097_FS	GTCGGAGCTTCTCCTCCTC
as-um02125_FS	CATCCTGGGGAGGACCTATC
as-um02150_FS	GGTGCTCGGTCACGAGTC
as-um02151_FS	CATTGGTGTTAGCAACTACGG
as-um02168.2_FS	CCTGCCATTCTACGAAAAGG
as-um02169_FS	GACACCCGTCTACATCCCTG
as-um02375_FS	TCTCTCGTGCTTCGGTGAC
as-um02403.2_FS	CGCAAGACAACGTTGAAGAC
as-um02523_FS	ACAAGTCAACCGTCCTCGTC
as-um02794_FS	TGCAAGTGCAGACACTGATG
as-um02803_FS	GTTGGCAGATGTGTGGTGAC
as-um03038_FS	TCAAGAAGGTCAAAGGGTGG
as-um03097_FS	GAGCACACCGACACCAGAC
as-um03263_FS	ACTTCCGATGGTGTCTCAGC
as-um03351_FS	GAAAAGGAGACGCGTATTGC
as-um03413_FS	TGGAAGTTCGATCTGCAGTG
as-um03618_FS	CGAATTCAATCGATGTCTGG
as-um03630_FS	AGCTTGCACTTTACTTGGGG
as-um03645_FS	CATGTACTTTGCCTATGCGG
as-um03798_FS	ACATCATCGCTTGTGGCTTC
as-um03841_FS	ATGTTCCTGGAGAATCTCGG
as-um03898_FS	GATGTATTTCTGTTCGGCGG
as-um03914_FS	GCATATTCTCTCGTGCAAAGG
as-um04059_FS	GTTGCTGGATTTCGTCTTGC
as-um04061_FS	CGGTACCCAAGATCCTTGTC
as-um04327_FS	TCGATTGATGAACGATGTCAC
as-um04361_FS	CTGATTTCATGCAAGACGGC
as-um04381_FS	AAACATCGACACCAAGGAGG
as-um04397_FS	CGTTCGAGTACGTCAAGCAG
as-um04466_FS	TTTCAAGTGCCAAACCTGTTC
as-um04495_FS	CGAATGGATCGAATGGCTC
as-um04654_FS	CTGGAAGCTACCACAGGTGC
as-um04878_FS	CTTCAGAGCAGGCAGAGCAG
as-um04959_FS	GCTAAGCAAGCGGTCAACTG
as-um05014_FS	TGAGCTTCCCCAACTACCAC

as-um05126_FS	GTGTTATGCCAAGCGAATTG
as-um05131_FS	GATGTGGAATTTCCTGGTGG
as-um05271_FS	ATATCACCGCTGCAATCCAC
as-um05363_FS	ACGAGGTCCGCAACAGAG
as-um05454_FS	GCGACGTTTACTACGAAGGC
as-um05564_FS	GTGAGCGGTCGTCTTGGTC
as-um05702_FS	AAAAATGCAAGGGTGTCTGC
as-um05725_FS	GCCCTTTTTCACAAGTCGAG
as-um05737_FS	GTCAAATCTCGGATGGCTTG
as-um05880_FS	TGAATACGCACATCCTCTGG
as-um05989_FS	GCTATTCCTGCTGAGCCTTG
as-um06045_FS	AGGACGTATTGCAGGACAGC
as-um06117_FS	AGATGGGTATCGTCGGTCTG
as-um06130_FS	CGTCTCGCTACCAATATCTCG
as-um06164_FS	GAGATCTACGCCAAAAAGCG
as-um06188_FS	GTACTTTCTCAAGCCTGCCG
as-um06281_FS	CAAGCTCAAAGTGCTCATGG
as-um06285_FS	TGATGCGACTTGGCAGTATC
as-um10002_FS	ATTGTCCTTGGCAACATTCC
as-um10018_FS	GCAACCTTTTCTTCAAACCG
as-um10027_FS	AAGCTTTAGCGAGCATCCAG
as-um10062_FS	AAGGTGTTGATTGTGGGAGG
as-um10078_FS	AAATAGCCAGGATGAGGTGG
as-um10213_FS	AACGCTGTCCGCACCTAC
as-um10671_FS	GTCACCGGCTACGAATCTG
as-um10751_FS	GCACTACTACCGGGAAATCG
as-um10792_FS	AAGCTGCTGCTAACCGTCTC
as-um10881_FS	GAAGTTTCCCCTTGCCCTC
as-um10946_FS	TAGCGCTAGCTGCCTACAAG
as-um11009_FS	CGACTTTGACTCGTACTCGC
as-um11010_FS	TTACCGATGACGACCATGAC
as-um11585_FS	ATGTCTTGTACTGGCGAGGC
as-um11590_FS	TCACACTTCGTGAGTGGCAG
as-um11603_FS	ACATCAGAACCAAGAACGCC
as-um11644_FS	TCGAAGTGGACTGTCTGCTG
as-um11668_FS	TTCCACAGGGCTTCGTTTAC
as-um11696_FS	TTCAAGGTTAAGACCACGGC
as-um11941_FS	CGACAATGCATTTACCTCGC
as-um12087_FS	TCAAGCCAGGTTACCTCACG
as-um12232_FS	ATCAAGCTACCAGCAAAGCG
**RT-PCR**	
umgapd_F	CATAATGTCTCAGGTCAACATCG
umgapd_R	GGATGTTGGAGGGGTCCT
um00047_F	CCTTGACAGTCTCAGCCTCG
um00047_R	GATCTCCTGCTCCACCTGTC
um00067_F	GCCTGCTCATCTCACAACTG
um00067_R	GTAAACTCTTTTGCTGCGCC
um00133_F	AACGAGATCAATGAGGGTCG
um00133_R	CTACCCGACACCTTTCTTGC
um00169_F	TCAGTCAGATCGAAAAGGGC
um00169_R	GGAATGTCGGGTATGTCGTC
um00329_F	GAGCCTTGTGTTGCTTGCTC
um00329_R	GAGCAGGAGACCGTGAAAAG
um00712_F	ATCTGGCTCACTTACCGACC
um00712_R	GTGCATCGTCGTAAATACCG

um00751_F	CCGTAAAGTTTGCAATCATCC
um00751_R	GGTCTTTTGAGCCTCTGACG
um00784.2_F	TCGGACGACACTTTGACAAG
um00784.2_R	TAATGGATCAATGTCGACGC
um00842_F	ATCACACCCTTTTGCGTGTC
um00842_R	GGCAACAAAGATGGCACTG
um00872_F	CACCACCAACCTCCTTCTTC
um00872_R	GCAATGTCGGTATCCGAGTC
um00913_F	CTCTACGTAGCGCAGCTTCC
um00913_R	TGAAACATGTCGTTCATCGG
um00947_F	CGGATAAGTCAGCTTCACCC
um00947_R	CCTTGCGGATATTGAGCTTC
um01110_F	AGAAGAACGAGGATGCATGG
um01110_R	TCCGACGTATTGGCTACCTC
um01252_F	CTGAACGTCGCAAGACACTG
um01252_R	TGGTTCTTGACCATGCTACG
um01439_F	TTCTCCTTGTCAAAAGATGCG
um01439_R	ATCAATAAAGCCTTGGGCTG
um01495_F	GATGCACAGTCGGTATGCAC
um01495_R	ACTGAAGCATGAAGCGTGTC
um01627_F	CACCAAGTCCCTCAAGGATG
um01627_R	GGTCTCAGAGTCGAGAACGG
um01668_F	TTCTTAACGCAGAGCCTTGG
um01668_R	CCCACACTAACTCCATTCGG
um01670_F	CTCAAAAGCGGAGAGGTGTC
um01670_R	GGTACCAGAATCGGAAGGTG
um01796_F	CATGAATCACAGCCCCTTG
um01796_R	CGGCAAATACTTGGTTGTTG
um02075_F	GTCGCGAGGATCAAGGTC
um02075_R	GTAACATCCCTGTTGCAGCC
um02097_F	ACAATATGGTCAACCGGCTC
um02097_R	GAGCAATCGACTTCTGTCGG
um02114_F	AGGGTCACCTTCACAAGTGC
um02114_R	ACGTGTAACGACTGCGAGTG
um02125_F	CCAATGTTCTTTCCTCCTCG
um02125_R	GGTCGGAAGCAGATTTGAAG
um02150_F	GTGTCACCACGCACAAGG
um02150_R	CTCGAGAGACATGTGATCGG
um02151_F	CGAGATTCACCCTTGGTGTC
um02151_R	GAAGCCCTTTTGCAACGAC
um02151_NO_F	ATCTGCGTACCCTTTGTTGG
um02151_NO_R	TGATCTTGGTGGTGACGAAC
um02168.2_F	AGAAATGCACCACTATCGGC
um02168.2_R	GGATAGGTGTCTTTCCGTCG
um02169_F	GCGAGCTGGAACTAGGTGTC
um02169_R	TGGGAAATCCTTTGAAGACG
um02375_F	CCGTGGACCCTAACGACTAC
um02375_R	TCTCGTCTCGGATCCATTTC
um02403.2_F	TTTGAGCGAATGCAACTGTC
um02403.2_R	TTGGTAAGGTATCGATCGGC
um02523_F	ATGCGACCATGTATTGGGAC
um02523_R	GCTGCTGACAGCTACACTGG
um02794_F	GACGTTCTGATTGGGAATGG
um02794_R	TCGATCAAACCTGCTGTCTG
um02803_F	TATGGTCGAGTCGAGGTTCG
um02803_R	GAGTCCAATCGAGTCCGAAG

um03038_F	GGCTGGGTATGAGCAGGAC
um03038_R	CAGAGCCAGGTTCGACTTTG
um03097_F	CAAGCTTTGAACCCCAACG
um03097_R	GCCTGCTCTTATCGCAACTC
um03263_F	ACTTGCAAATCGTTGCACTG
um03263_R	TAAACTTGCGTGTCGTGTCC
um03351_F	GCCGACTGTGTATCGAAGTG
um03351_R	GCAAACGATGCAACTTGAAC
um03413_F	TCGGTAGATCCGGTGTTAGC
um03413_R	ATCTTCAACACATACGCCCC
um03618_F	TGCGACACTATGCTCGAATC
um03618_R	GCGTAGTCCTTATCGATGCC
um03630_F	TCCAACTCGCTTTCCAAGTC
um03630_R	AGCCAAAGCTGACCGTGTAG
um03645_F	ACTGGACCACAGACTCGACC
um03645_R	CAGAGTCCTTGGGCATCATC
um03798_F	CAACATTGTCCGCATCTCAC
um03798_R	ACGCGTGTTTGTTGATCTTG
um03841_F	GTCGGTACAAGACCTGCTGC
um03841_R	CCTGATCAATGGTTGGTCG
um03898_F	CAGCTGGCCTACTATCTGGG
um03898_R	TCGCAAGCTATCTAGGTCCG
um03914_F	CAGGTATACGGGAGGCAGTG
um03914_R	ACAGACGTTCTGGCGTATCC
um04059_F	CGTTACGATCCGAACGGTAG
um04059_R	CCATGTTGCAGGCTAGATTTC
um04061_F	AATGGGTGCTGTCGAACTTG
um04061_R	TCTGAGGTCCCGTAAAGCAC
um04327_F	AGTTGCATACCAAGCAGTCG
um04327_R	ATGAACGCCTCGGTAGTTTC
um04361_F	GCTCTTTCGAAATCGTCCAG
um04361_R	ATTCTTCAACCCAACAACGC
um04381_F	CAAAGGTGACGGTGACAATG
um04381_R	CTCTTCGTCGGCAATCATTC
um04397_F	TGCTCATGACCATTCTCACC
um04397_R	AAGGCGTCAATGTGGTTTTG
um04466_F	TCTACATTCTCGGACGACGC
um04466_R	TTTCCGAGAAAGACCAGAGC
um04495_F	CACAAGTACCTCATCCAGCG
um04495_R	GAGTGAGGAAGCAAGGATGC
um04654_F	TTTGATGGAGACAGAGGATGG
um04654_R	TCCAGGCTTTCGCTACTCTC
um04878_F	GAGCTTCGTGACATGGTGG
um04878_R	CTTCTTCTTCTTGGCGCTTC
um04959_F	CGTGCCCCATGTTTACCTAC
um04959_R	GATCGAAGCGTCTTCCTCTG
um05014_F	CCGACATCTTTCCCAGTACG
um05014_R	AGTCGTAGGTGGTGCTGGAC
um05126_F	GGGATTGATGTGGGATTCTG
um05126_R	ATTTGGGCTGATCAGTTTGC
um05131_F	TACGAGTGCTGCGTACCTTG
um05131_R	TGATCTCTTGACGTGCATCC
um05271_F	CTCCAGTTCCAGCTCGTCTC
um05271_R	TACCCATAGCAAAAGTGCCC
um05363_F	AAAGACCGAAGGTCCGGTAG
um05363_R	TGGTAAGAGTCCTCATCGTCG

um05454_F	TATCCGCATCTCCAAATTCC
um05454_R	ACTGCAGACCGGATACGTCC
um05564_F	CCAAGCTGTACTTGGTGGTG
um05564_R	TCGCCTCTTCTTTTGTCTGC
um05702_F	ATCGGCAACTACAAGCATCC
um05702_R	ATTTCCAGTGGTATCAGCCG
um05725_F	TGGCACAGAACGAGTACCAC
um05725_R	ATCACGTTGAAAGGAAAGCC
um05737_F	AATCATTCGAACAACACGAGG
um05737_R	ATGTCGATGTTGTGCAGCTC
um05880_F	CGTTCACCAAGGACCTTCAC
um05880_R	GGAACGACGTCGAGTAGCAG
um05989_F	ATTGGAGTGTCTCAGGGTGG
um05989_R	AGCAAGGAGCAGATGACGAC
um06045_F	AGAACTGCTTCCAAGATGGC
um06045_R	GTCTGCCGTAGATAGGCTGC
um06117_F	CATTGCGAAATGTGACTTGG
um06117_R	TGCTCAAGAAAGCGTTACCC
um06130_F	ATGCATTGGGCGAATTTTAC
um06130_R	ATGCAAGATACGAAATCCGC
um06164_F	CTCTGCACGACATCCACAAC
um06164_R	CGTTCGAGCCACGATACC
um06188_F	TGAGCAGTGCATCTACTGGG
um06188_R	TTGACATTGTCTTTGGGCAC
um06281_F	GGCATGTCGAGACGTTGTC
um06281_R	AGCCACAGCAAATTTCAAGG
um06285_F	GCGTGTTTGGTGAAACAGTG
um06285_R	GTGTCGACGCCAAAGATACC
um10002_F	TGGCTCACACCAAGAGTACG
um10002_R	TTCAAACACGATACGCGAAC
um10018_F	CTTCGTTTGCCAAAGTGCTC
um10018_R	GTCACCCATTCTCCTTGGTG
um10027_F	GTGCTGCTGAAACAGGTGG
um10027_R	AACCTGAGTGATTCGCTTGG
um10062_F	CATCCCACGCAATACCATC
um10062_R	CGGCTTCGTAGTGGATTTTC
um10078_F	ACACTGACGGCATGCTACC
um10078_R	TCACAAGGTCAACCAACCAG
um10213_F	GGTCAGCTCCATCCTTGAAC
um10213_R	TCAATGGGGTTACCAAGAGC
um10671_F	TCGGCTACCTGTGATGGTTC
um10671_R	TGTGGTCGAAAATGGTGTTG
um10751_F	TTCGACTACTGTGCAGGGTG
um10751_R	TCCAGAACTGACGGAGGAAC
um10792_F	CTTTCCACATGCAGCAACAC
um10792_R	GTCATCGATCACCTTTTCGG
um10881_F	GCAAGAGCTACAGCGCATC
um10881_R	TCCGCCAGTGTATCCAGAAG
um10946_F	GCGACTCTTTGGATGAGGAG
um10946_R	ATAGCCACGTATCGTCCTGC
um11009_F	TTTCACATACCGCCTCATTG
um11009_R	GCCAGCTGAAAGAGGTGAAC
um11010_F	GCCTCGATCTCTTTGTCTGG
um11010_R	ATACCGTCTCACCCCACAAC
um11585_F	TACGTGAAGCAGCCAACAAG
um11585_R	AAAGACAAGCGAGTTGGAGC

um11590_F	CAAGGCTCAGCTCAAGGC
um11590_R	ATACCCTCAACCTGTCCGTG
um11603_F	TCACTCTCAAGAACCCTGCC
um11603_R	ATCATCGAAAGGTCACCACC
um11644_F	TTGCGATCGGTGATATTCAG
um11644_R	GACCACCTTGTCACCTGGAG
um11668_F	ATCATCGCTATCACGCACAG
um11668_R	ATCTGATCGTCCGTTTACGC
um11696_F	CAGTCGACCATCATCACACC
um11696_R	CCTCCTTGGCTTTCTGAGTG
um11941_F	AGATGAAACGCATCGAGCAG
um11941_R	CCATCATCTCGTTGATGAGC
um12087_F	CGAAATCCACCTCTTTCTCG
um12087_R	CTCTTTGCCTGCTCCTCATC
um12232_F	TCCGATCCATCTGGCATATC
um12232_R	ATGACCGACTTGTTCCAAGG

Tagged strand-specific first strand synthesis

um02151_FS+Tag_OL	cgaggatcatggtggcgaataaCCTGATCCTTCGCATCTAGCTTC
um02151_FS+Tag_NO	cgaggatcatggtggcgaataaGTAGCCATCCAGATTGATTTTTG
as-um02151_FS+Tag_OL	cgaggatcatggtggcgaataaCATTGGTGTTAGCAACTACGGAG

Tagged RT-qPCR

Tagged_Primer	cgaggatcatggtggcgaataa
um02151_OL_q	CGGTCAACCAGATCGAGATTC
um02151_NO_q	GCAATCTGCGTACCCTTTGTTGG
as-um02151_OL_q	GAAGCCCTTTTGCAACGAC

RT-qPCR TaqMan MGB Probes

umgapd_INT_VIC	VIC-ATCAACGGCTTCGGTC
um02151_OL_6FAM	6FAM-AGCGACCCATTGTC
um02151_NO_6FAM	6FAM-CGAGTCGGGCGTCG

RT-qPCR Control

umgapd_qF	CTTCCATAATGTCTCAGGTCAACATCG
umgapd_qR	GGATGTTGGAGGGGTCCTTCTC

RLM-RACE

as-um02151_5'R_Outer	CATTGGTGTTAGCAACTACGG
as-um02151_5'R_Inner	CGGTCAACCAGATCGAGATTC
as-um02151_3'R_Inner	GAAGCCCTTTTGCAACGAC
as-um02151_3'R_Outer	CCTGATCCTTCGCATCTAGC
as-um02150_5'R_Outer	GGTGCTCGGTCACGAGTC
as-um02150_5'R_Inner	GTGTCACCACGCACAAGG
as-um02150_3'R_Inner	CTCGAGAGACATGTGATCGG
as-um02150_3'R_Outer	GTAGCAGCGAACTCAAGATGG

Ustilago hordei

Strand-specific first strand synthesis

uhor_01678_FS	TGCAGACTGTTCATCACTTCG
uhor_03521_FS	AACCTCACAAAGTCCCCAATC
uhor_03549_FS	CAACACGCTTAGCGGAGTAAG
uhor_03676_FS+Tag	cgaggatcatggtggcgaataaCCAAATCCTTCGCATCCAACTTG
uhor_05387_FS	TCGTTTGACAAATCCTGATCC
uhor_08740_FS	CGTTCAGGACTACCCCTCTTC
as-uhor_01678_FS	CGTGCAGAAGAACGAGGAG

as-uhor_03521_FS	CTCATGAAAGGAAGCGAACTG
as-uhor_03549_FS	CAAGTCAATATTCGCCAAACC
as-uhor_03676_FS+Tag	cgaggatcatggtggcgaataaGATCGGTGTTAGCAACTATGGTC
as-uhor_05387_FS	GCCTCCAAGAAAGAAAAGCTC
as-uhor_08740_FS	AAAAGGCTATCCCAATCAACC
**RT-PCR**	
uhor_01678_F	ACTACAATGTGGGCAAGAACG
uhor_01678_R	CTTCGAACGAGTGGATTGTTG
uhor_03521_F	TCCAATGCACCTTCCTACTTG
uhor_03521_R	CCCATCCTTGCCATACCTTAG
uhor_03549_F	AATTCGCTGCTACTCCTCCTC
uhor_03549_R	CTTTCACCTGACCACGCATAG
uhor_03676_F	CGAACTTCACCCTTGGTGTC
uhor_03676_R	CAAACCCTTTCTGCAACGAC
uhor_05387_F	GACAAGCACGGATACTTCAGC
uhor_05387_R	GACTCCTTCATGACGTCGAAC
uhor_08740_F	GGAATCACCGGCTATGTCAA
uhor_08740_R	AGCAGCACCGATCTATGAGG
uhor_12743_INT_F	CCCAGGTCAACATCGGTATC
uhor_12743_INT_R	GACACCGCAAACATACATGG
uhor_08874_F	AGGCTTGCTCTTCCAGACAG
uhor_08874_R	AAATGTGTTCCCACCAAAGTG
uhor_01631_F	GCTGTTCTTCCTGGTGTCAAC
uhor_01631_R	CTGCCCTCCACTTCATCATC
uhor_06256_F	ACCTTCCTCCTGGCATCTG
uhor_06256_R	ATCTCGGGCATCTCAATGTC
uhor_08937_F	AAGGCGTGCTGGCTAAGAC
uhor_08937_R	GGGTTTGAATGGTTGACTTCC
**RLM-RACE**	
as-uhor_03676_5'R_Outer	GATCGGTGTTAGCAACTATGGTC
as-uhor_03676_5'R_Inner	CGAACTTCACCCTTGGTGTC
as-uhor_03676_3'R_Inner	CAAACCCTTTCTGCAACGAC
as-uhor_03676_3'R_Outer	CCAAATCCTTCGCATCCAACTTG

[a]Lower-case letters in primer sequences represent nucleotides not complementary to the *U. maydis* genome.
[b]Restriction endonuclease recognition sites are underlined.

Table S6. Plasmids used in this study.

Plasmid	Source	Relevant primers	Restriction endonucleases used in cloning	Antisense transcript length (nt)
**Antisense transcript expression analysis**				
pCM768	Kojic and Holloman, 2000			
pCMas-um02114	This work	as-um02114_F_BamHI, as-um02114_R_HindIII	*Bam*HI and *Hind*III	1168
pCMas-um02125	This work	as-um02125_F_SphI, as-um02125_R_HindIII	*Sph*I and *Hind*III	1436
pCMas-um02150	This work	as-um02150_F_SphI, as-um02150_R_HindIII	*Sph*I and *Hind*III	648
pCMas-um02151	This work	as-um02151_F_BamHI, as-um02151_R_HindIII	*Bam*HI and *Hind*III	458
pCMas-um10002	This work	as-um10002_F_HindIII, as-um10002_R_HindIII	*Hind*III	1118
pCMas-um12232	This work	as-um12232_F_BglII, as-um12232_R_BglII	*Bgl*II	2057
**Deletion analysis**				
pMF1-h	Brachmann *et al.*, 2004			
pCR-XL-TOPO-Δum02151	This work	um02151_Nested_F, um02151_Nested_R	*Sfi*I	
pCR-XL-TOPO-$\Delta P_{as\text{-}um02151}$	This work	pA_Nested_F, pA_Nested_R	*Sfi*I	

nt: nucleotides.

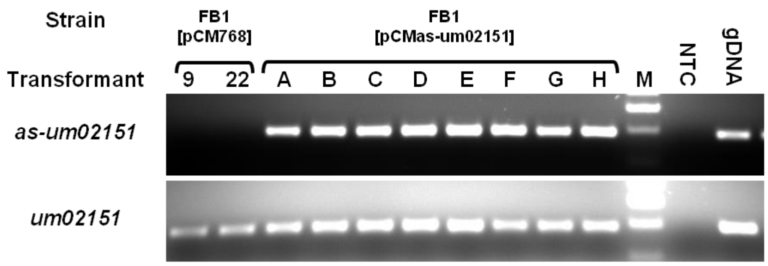

Fig. S1. Relative levels of *as-um02151* and *um02151* in haploid transformants. RT-PCR confirms *as-um02151* expression and reveals enrichment of *um02151* levels in vector transformed haploid strains expressing *as-um02151* (FB1[pCMas-um02151]), relative to independent empty-vector transformed strains (FB1[pCM768]). For each sample, internal *umgapd* levels were used as a reference transcript (data not shown).

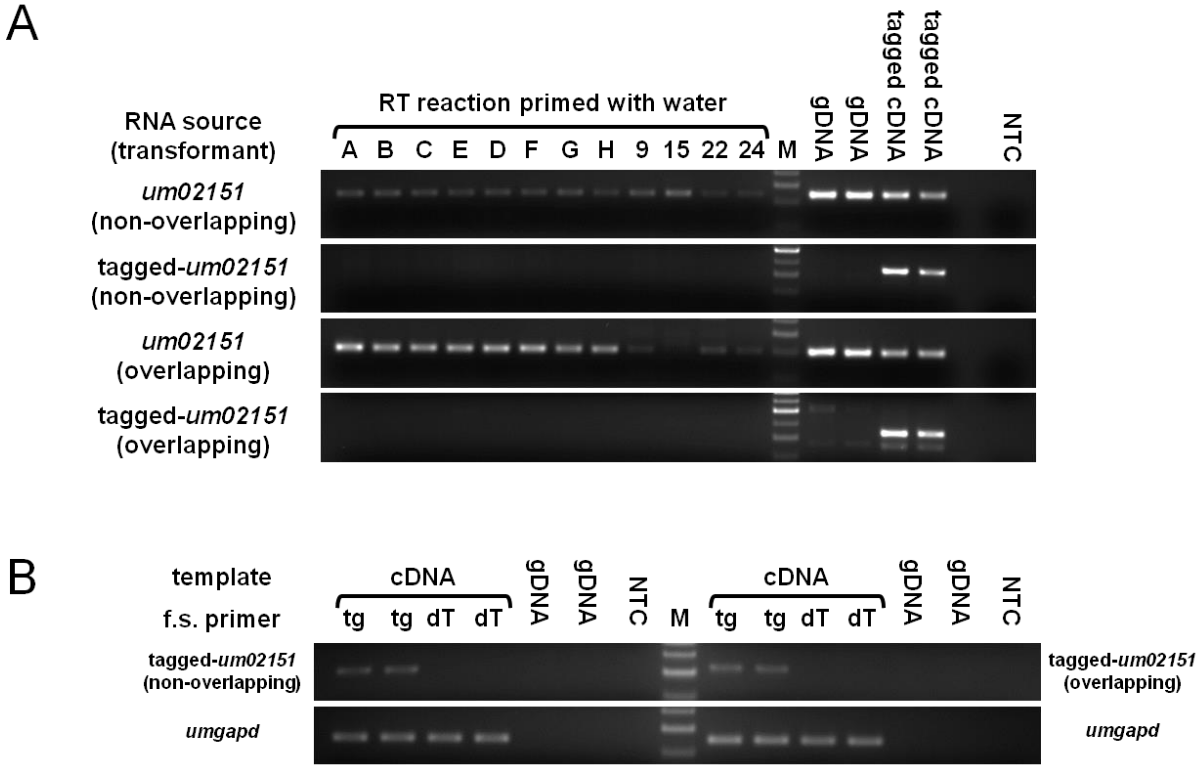

Fig. S2. Tagged RT-PCRs decreased background amplification observed in conventional RT-PCR.

A. Tagged RT-PCR eliminates false-priming. To test the specificity of tagged RT-PCRs, RNA was isolated from 8 independent FB1[pCMas-um02151] strains (A-H), and 4 independent FB1[pCM768] strains (9-24). Reverse transcriptase reactions were primed with water (A-H, 9-24), creating 'false-primed' cDNA. Conventional PCRs (using a forward and reverse primer), or tagged *um02151*-specific PCRs (using the tagged primer system) were conducted to amplify the non-overlapping sense-antisense region of the *um02151* transcript, or the overlapping sense-antisense region of the *um02151* transcript. The following were included as PCR controls: genomic DNA (gDNA), tagged cDNA, or no template (NTC). A DNA marker (M) was also included.

B. Tagged qPCR is highly specific. The tagged primer system was used in conjunction with TaqMan qPCR. The primers used in first strand synthesis (f.s.) were: tagged strand-specific (tg), or oligo-$(dT)_{16}$ (dT). An internal *umgapd*-specific primer was added to the tagged strand-specific reaction to assess relative amounts of mRNA. The resulting cDNAs were used as templates for tagged qPCR. The following were included as qPCR controls: genomic DNA (gDNA), or no template (NTC). A DNA marker (M) was also included.
